# A shrinkage-based statistical method for testing group mean differences in quantitative bottom-up proteomics

**DOI:** 10.1186/s12859-025-06275-1

**Published:** 2025-10-31

**Authors:** Namgil Lee, Hojin Yoo, Juhyoung Kim, Heejung Yang

**Affiliations:** 1 Department of Information Statistics, Kangwon National University, Gangwondaehak-gil 1, Chuncheon, Gangwon 24341 Republic of Korea; 2 Bionsight, Inc., Gangwondaehak-gil 1, Chuncheon, Gangwon 24341 Republic of Korea; 3 Department of Pharmacy, Kangwon National University, Gangwondaehak-gil 1, Chuncheon, Gangwon 24341 Republic of Korea

**Keywords:** Differential analysis, Ionization efficiency, Shrinkage estimation, Tandem mass spectrometry

## Abstract

**Background:**

In bottom-up proteomics using data-independent acquisition mass spectrometry (DIA-MS), quantitative measurements are obtained following multiple steps of protein fragmentation and ionization, which introduces cumulative errors and impairs the effectiveness of classical statistical methods. This study proposes an alternative statistical approach for testing group mean differences at the peptide level in quantitative bottom-up proteomics.

**Results:**

We present a novel probabilistic graphical model, that accounts for the non-normality of empirical distributions and the correlations between fragment ion quantities. Based on the model, we propose a new statistical method that improves upon the classical feature-based approach by incorporating distribution-free shrinkage estimation of covariance matrices and bootstrap-based estimation of degrees-of-freedom. Simulated experiments demonstrate that the proposed method outperforms the four most widely used classical methods in terms of specificity, sensitivity, and accuracy, particularly when the data distribution closely resembles real MS data, and under conditions of small sample sizes. Numerical analysis of real quantitative tandem mass spectrometry data reveals that the proposed method effectively identifies candidate peptides exhibiting changes in mean quantity following treatment with the kinase inhibitor Staurosporine.

**Conclusions:**

The proposed statistical method offers an effective alternative to classical approaches for differential analysis of peptides in quantitative bottom-up proteomics using DIA-MS. The R software package MDstatsDIAMS is available at https://github.com/namgillee/MDstatsDIAMS.

## Background

Due to technological advances in high-resolution mass spectrometry (MS), quantitative measurements of proteins and peptides across multiple conditions have increased [1]. Proteomic experiments are often conducted using complex experimental designs that involve numerous conditions and replicates, with the primary focus of the analysis being the detection of quantitative changes across conditions [2]. As the size and complexity of liquid chromatography coupled with tandem mass spectrometry (LC-MS/MS) data increase, there is a greater need for statistical methodologies to detect differentially abundant proteins and peptides [3].

Differential expression analysis workflows for proteomic data consist of multiple stages [4]. At the first stage, analysis software platforms such as Spectronaut [5], MaxQuant [6], and Skyline [7] perform peptide identification and quantification by their own complex workflows. The rest of the stages is often referred to as a downstream analysis or post-processing, and it includes the matrix representation stage, normalization stage, imputation stage, and statistical analysis stage [4]. For the final statistical analysis stage, classical statistical methodologies such as the analysis of variance (ANOVA) [8], *t* test [9], and linear mixed model [3, 10–12] have been widely used. But classical methodologies are often based on strong distributional assumptions, such as normality or independence, which are inconsistent with the characteristics of MS data acquisition techniques in recent bottom-up proteomics.

This study proposes an alternative statistical method for differential expression analysis at the peptide level, which post-processes quantification reports generated by analysis software platforms. Especially, the proposed method aims to analyze data-independent acquisition mass spectrometry (DIA-MS) data in bottom-up proteomics [13], where spectral features correspond to both precursor peptides and fragment ions.

In general, statistical methodologies for differential expression analysis can be categorized into two-step methods or feature-based methods [3]. In two-step methods, all feature intensities of each protein or peptide in a run are summarized, and then statistical analysis is performed on the summarized quantities. Feature-based methods conduct statistical analysis directly on the feature-level quantities. For example, ROTS [14] takes protein-level summarized quantities and selects an optimal test statistic among a family of modified *t*-statistics. MSqRob [11, 12] and MSstats [3] can perform both feature-based and two-step based analyses at the protein-level. For feature-based analysis, they take quantified features as input and use a linear mixed model for differential analysis. However, two-step methods tend to yield reduced sensitivity compared to feature-based methods, especially due to small sample sizes and bias [15]. On the other hand, feature-based methods are prone to underestimating between-sample variability and the correlation between features of the same protein (or peptide), which results in an inaccurate estimation of the degrees-of-freedom [12, 15].

In this study, we propose a novel hierarchical probabilistic graphical model to address the characteristics of real-world MS data distributions, including non-normality and the correlation among fragment ion quantities. The proposed model is a generative model that represents hierarchically structured LC-MS/MS data. In this model, two types of ionization efficiency are introduced as hidden variables for both the MS1 and MS2 spectra. Moreover, we propose a new *t* test statistic for detecting group mean differences. The proposed test statistic enhances a classical feature-based test statistic by addressing the random variation and correlation of ionization efficiencies. Specifically, covariances between fragment ion quantities are estimated using a James-Stein-type distribution-free shrinkage estimation method [16, 17], which can address the issue of reduced sensitivity due to small sample sizes. A bootstrap approach is suggested to accurately estimate the degrees-of-freedom of the proposed test statistic [18].

In principle, given a sample covariance $S_{ij}$ between two random variables $X_i$ and $X_j$ (e.g., representing fragment ion quantities), a James-Stein type shrinkage estimator of the covariance $S_{ij}^*$ can be expressed as a linear combination:

1 $$\begin{aligned} S_{ij}^* = (1 - \lambda ) S_{ij} + \lambda T_{ij}, \end{aligned}$$

where $0\le \lambda \le 1$ denotes the shrinkage intensity and $T_{ij}$ is the shrinkage target. When $\lambda = 1$, the shrinkage estimator reduces to the target $T_{ij}$, and when $\lambda = 0$, it coincides with the sample covariance $S_{ij}$. Although the sample covariance is unbiased, it suffers from high variance for small sample sizes or high dimensionality, resulting in low estimation accuracy. By contrast, the shrinkage target $T_{ij}$ is typically set to have low variance, such as a constant value. By selecting an optimal shrinkage intensity, the shrinkage estimator achieves improved accuracy through a balance between bias and variance.

Through the analysis of real MS data, we show that the proposed model effectively captures the actual data distribution, especially the variability and correlations in ionization efficiency. We also provide estimated hyperparameter values based on this real MS data. Simulated experiments confirm that the proposed test statistic is asymptotically consistent, following a Student’s *t*-distribution with accurately estimated degrees-of-freedom. Numerical experiments using both simulated and real DIA-MS data demonstrate that the proposed method outperforms two classical statistical methods, paired *t* test and independent samples *t* test, and two modern statistical methods, ROTS [14] and MSstatsLiP [19], in terms of specificity, sensitivity, accuracy, and area under the receiver operating characteristic curve (AUC), particularly when the hyperparameter values closely match the real MS data distribution.

The remainder of this paper is organized as follows. Section 2 describes the proposed hierarchical graphical model and the shrinkage-based statistical test method. Section 3 presents numerical experiments that compare the performance of the shrinkage-based statistical test method and the other classical methods by using simulated data. In addition, we analyze real DIA-MS data from HeLa cells treated with the kinase inhibitor Staurosporine at multiple doses to identify peptides with significant changes in mean quantity. Discussion and conclusions are provided in Sect. 4.

## Methods

### Hierarchical probabilistic graphical model for bottom-up proteomics

Consider an experiment with multiple conditions, $c=1,2,\ldots ,C$, and a few technical replicates, $r=1,2,\ldots ,R_c$, for each condition *c*. In bottom-up proteomics, peptides can be represented in three different forms: stripped sequences, modified sequences, and precursors. Among these, a precursor is the most specific representation, defined by an amino acid sequence with possible modifications and a specific charge state. A modified sequence retains the amino acid sequence and its modifications but ignores the charge state. A stripped sequence includes only the amino acid sequence, ignoring both modifications and charge state. In this study, each peptide, denoted as $p = 1, 2, \ldots , P$, refers to a precursor-level peptide, i.e., the most detailed form of representation. As a result, peptides with the same amino acid sequence but different modifications or charge states are treated as distinct precursors. Table 1 summarizes the mathematical notations used in this paper.

**Table 1:** Mathematical notations

Notation	Description
$c=1,2,\ldots ,C$	Condition
$r=1,2,\ldots ,R_c$	Replicate at condition *c*
$p=1,2,\ldots ,P$	Precursor peptide
$i=1,2,\ldots ,I$	Fragment ion
$f_{cir}^p$, $x_{cir}^p$ ($= \log _{10} f_{cir}^p$)	Quantity of fragment ion *i*, and its $\log _{10}$-transformation
$q_{cr}^p$, $y_{cr}^p$ ($= \log _{10} q_{cr}^p$)	Peptide quantity of precursor *p*, and its $\log _{10}$-transformation
$w_{cir}^p$, $\xi _{cir}^p$ ($= \log _{10} w_{cir}^p$)	Ionization efficiency for fragment ion *i*, and its $\log _{10}$-transformation.
$u_{c}^p$, $\zeta _{c}^p$ ($= \log _{10} u_{c}^p$)	Data acquisition rate for peptide *p*, and its $\log _{10}$-transformation
$\Delta \xi _{ir}^p$ ($= \xi _{1ir}^p - \xi _{2ir}^p$)	Difference between $\log _{10}$-transformed ionization efficiencies
$e_{cr}^p$	Sampling error

**Fig. 1:**
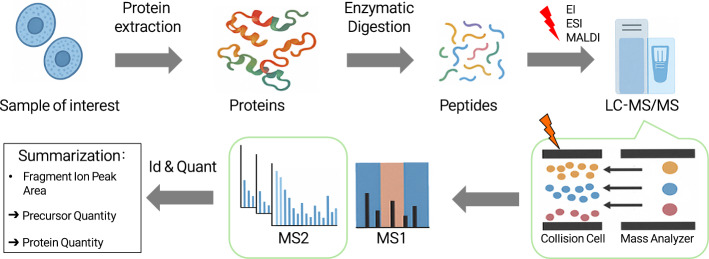
Workflow for quantification in bottom-up proteomics using DIA-MS. The red lightning symbol represents peptide ionization by methods such as Electron Ionization (EI), Electrospray Ionization (ESI), or Matrix-Assisted Laser Desorption Ionization (MALDI). The orange lightning symbol with a black outline indicates the fragmentation of these ionized peptides (precursors) into fragment ions via Collision Induced Dissociation (CID) in the collision cell of the mass spectrometer. The fragment ions are then analyzed to obtain MS2 spectra, which are quantified based on their peak area

Figure 1 illustrates the workflow for peptide quantification in bottom-up proteomics using DIA-MS. In this approach, proteins are enzymatically digested into peptides and the resulting peptides are ionized with specific charge states as they are injected into the mass spectrometer. While the mass spectra of the precursors are first measured in a survey scan, known as the MS1 spectrum, the precursors are fragmented in a collision cell, producing fragment ions whose spectra are collected as MS2 spectra. In DIA-MS, these MS2 spectra are used to quantify fragment ions, which are then aggregated to quantify their corresponding precursors, and further summarized to infer protein-level abundances. Due to the hierarchical nature of the data acquisition process, MS2 spectra are often subject to data loss, non-normal distributions, and inter-run dependences.

**Fig. 2:**
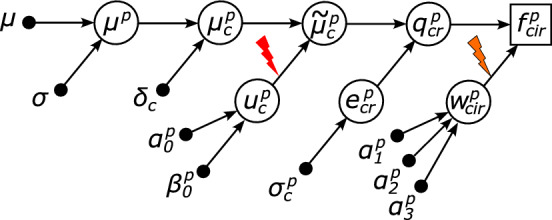
Graphical illustration of the hierarchical probabilistic graphical model for quantitative bottom-up proteomics. The squared node, $f_{cir}^p$, represents the quantity of a fragment ion, which is an observed variable. The circled nodes represent latent variables, and the black dots represent hyperparameters of the distributions that generate the latent variables. The red lightning symbol indicates peptide ionization and the orange lightning symbol with a black outline indicates the fragmentation of the ionized peptides; see Fig. 1. Mathematical notations and their descriptions are summarized in Table 1

We suggest a hierarchical probabilistic graphical model for simulating quantitative bottom-up proteomics, which is graphically illustrated in Fig. 2. In sequential window acquisition of all theoretical mass spectra (SWATH-MS) DIA, quantification is usually performed based on MS2 spectra [13, 20]. Let $f_{cir}^p$ denote the measured quantity of fragment ion *i* of peptide *p* under condition *c* and replicate *r*. A standard method for summarizing fragment ion quantities for a peptide is to sum the best *I* fragment ion quantities of the highest ranks, where typically $I\approx 3$ [21]. That is, $q_{cr}^p = \sum _{i=1}^I f_{cir}^p.$ The proportion of a selected fragment ion quantity $f_{cir}^p$ to the peptide quantity $q_{cr}^p$ can be parameterized as $w_{cir}^p = f_{cir}^p / q_{cr}^p$ for $i=1,2,\ldots ,I$, or equivalently,

2 $$\begin{aligned} f_{cir}^p = q_{cr}^p \cdot w_{cir}^p. \end{aligned}$$

The parameter $w_{cir}^p$ represents the probability that fragment ion *i* is detected and quantified, and it is referred to as the ionization efficiency.

The log-transformed peptide quantities, $\log _{10} q_{cr}^p$, $r=1,2,\ldots ,R_c$, are assumed to be independently and normally distributed with a mean of ${\tilde{\mu }}_c^p$ and a standard deviation of $\sigma _c^p$, as expressed by

3 $$\begin{aligned} \log _{10} q_{cr}^p = {\tilde{\mu }}_c^p + e_{cr}^p, \quad e_{cr}^p \sim \text {N}(0, (\sigma _c^p )^2). \end{aligned}$$

We assume that the mean of the peptide quantity, ${\tilde{\mu }}_c^p$, is a biased esimate of the true mean value, $\mu _c^p$, as

4 $$\begin{aligned} {\tilde{\mu }}_c^p = \mu _c^p + \log _{10} u_c^p. \end{aligned}$$

The random variable $u_c^p$, $0 < u_c^p \le 1$, denotes the proportion of the estimated mean peptide quantity to the true mean quantity as

5 $$\begin{aligned} 10^{{\tilde{\mu }}_c^p} = 10^{\mu _c^p} \cdot u_c^p. \end{aligned}$$

We note that the relative total amount of missing fragment ions can be expressed by $(10^{\mu _c^p} - 10^{{\tilde{\mu }}_c^p}) / 10^{\mu _c^p} = 1 - u_c^p$. This implies that $u_c^p$ corresponds to the ionization efficiency of the precursors, or in other words, the proportion of total measured fragment ion quantities. We will refer to $u_c^p$ as the data acquisition rate to distinguish it from the ionization efficiency $w_{cir}^p$.

To model uncertainty in data acquisition rate, $u_c^p$ is assumed to follow a beta distribution:

6 $$\begin{aligned} u_c^p \sim \text {Beta}(\alpha _0^p, \beta _0^p), \end{aligned}$$

where $\alpha _0 > 0$ and $\beta _0 > 0$ are the shape parameters. We assume that the $u_c^p$ are independent across conditions. Additionally, the ionization efficiencies $w_{cir}^p$, $i=1,2,\ldots ,I$, are assumed to follow a Dirichlet distribution:

7 $$\begin{aligned} (w_{c1r}^p, \ldots , w_{cIr}^p) \sim \text {Dirichlet}(\alpha _1^p, \ldots , \alpha _I^p), \end{aligned}$$

where $\alpha _1^p, \alpha _2^p, \ldots , \alpha _I^p > 0$ are the shape parameters. We assume that the $w_{cir}^p$ may be correlated across conditions with a correlation coefficient of $0 \le \rho ^p \le 1$, e.g., $\text {Corr}(w_{1ir}^p, w_{2ir}^p) = \rho ^p$. The assumption of a nonnegative correlation suggests that the ionization of the same fragment ion will be similar across conditions.

Each peptide is allowed to have a distinct mean quantity, $\mu _c^p$, and it is assumed that the mean quantities differ by a fixed constant between conditions, expressed as $\mu _c^p = \mu ^p + \delta _c$. We do not assume a specific probability distribution for $\mu ^p$ for theoretical analysis. However, for numerical simulation, the mean quantities are generated from a normal distribution with a mean of $\mu$ and a standard deviation of $\sigma$ as follows:

8 $$\begin{aligned} \mu ^p \sim \text {N}(\mu , \sigma ^2). \end{aligned}$$

### Shrinkage-based statistical test

Let $\mu _c^p$ denote the bias-corrected means of $\log _{10}$-transformed peptide quantities for conditions $c=1,2$, respectively; that is, $\mu _c^p = {\tilde{\mu }}_c^p - \log _{10} u_c^p$, $c=1,2.$ For comparing the mean values, the mean difference between the two groups can be written as:

9 $$\begin{aligned} L = \mu _1^p - \mu _2^p. \end{aligned}$$

Equation (9) can be extended to a linear combination of multiple mean values to address group comparisons involving two or more expected values, expressed as $L = \sum _c a_c \mu _c^p$ for any contrast coefficients $a_c,\ c=1,\ldots ,C,$ satisfying $\sum _c a_c = 0$. In general, a test statistic for testing group differences, e.g., $H_0: \mu _1^p = \mu _2^p \ \ \text {versus} \ \ H_A: \mu _1^p \ne \mu _2^p,$ can be expressed in the following form [3]: $t = {\hat{L}} / \text {SE}({\hat{L}}),$ where ${\hat{L}}$ is an estimate of *L* and $\text {SE}({\hat{L}})$ is the standard error of the estimate ${\hat{L}}$.

We propose an alternative test statistic and a bootstrap method for determining appropriate degrees-of-freedom. The alternative method is referred to as the shrinkage *t* test in this paper. The proposed statistical method is developed using fragment ion quantities, making it a feature-based methodology similar to the paired *t* test. However, the assumption of independence among fragment ions in the paired *t* test does not hold in real MS experiments. The hierarchical probabilistic graphical model described in Sect. 2.1 addresses this through the Dirichlet distribution in (7). The proposed method also tackles this issue by estimating the correlations directly.

The proposed test statistic can be expressed as

10 $$\begin{aligned} t_\text {shrink} = \frac{{\hat{L}}_\text {shrink}}{\text {SE}({\hat{L}}_\text {shrink})}, \end{aligned}$$

where the numerator is an estimate of the group mean difference, defined by

11 $$\begin{aligned} {\hat{L}}_\text {shrink} = \sum _{i=1}^I \left( {\bar{x}}_{1i}^p - {\bar{x}}_{2i}^p \right) , \end{aligned}$$

with ${\bar{x}}_{1i}^p$ and ${\bar{x}}_{2i}^p$ representing the sample means of $x_{1ir}^p$ and $x_{2ir}^p$, respectively. We note that the numerator is directly related to that of the paired *t* test statistic by ${\hat{L}}_\text {shrink} = I {\hat{L}}_\text {paired}$, where *I* denotes the number of fragment ions, in the case of an equal number of replicates, $R \equiv R_1 = R_2$. The denominator can be written as $\text {SE}({\hat{L}}_\text {shrink}) = ( \text {Var}({\hat{L}}_\text {shrink}) )^{1/2}$ by the definition of the standard error. Using (11) and the bilinearity of covariance, the variance term can be expanded as a sum of covariance terms: assuming that $R_1 = R_2 \equiv R$,

12 $$ \begin{aligned} {\text{Var}}(\hat{L}_{{{\text{shrink}}}} ) = & \frac{1}{{R^{2} }}\sum\limits_{{i = 1}}^{I} {\sum\limits_{{j = 1}}^{I} {\left( {\sum\limits_{{c = 1}}^{2} {\sum\limits_{{r = 1}}^{R} {\sum\limits_{{r^{\prime} = 1}}^{R} {{\text{Cov}}} } } (x_{{cir}}^{p} ,x_{{cjr^{\prime}}}^{p} ) + } \right.} } \\ & \left. { - \sum\limits_{{r_{1} = 1}}^{R} {\sum\limits_{{r_{2} = 1}}^{R} {{\text{Cov}}} } (x_{{1ir_{1} }}^{p} ,x_{{2jr_{2} }}^{p} ) - \sum\limits_{{r_{1} = 1}}^{{R_{1} }} {\sum\limits_{{r_{2} = 1}}^{{R_{2} }} {{\text{Cov}}} } (x_{{2ir_{2} }}^{p} ,x_{{1jr_{1} }}^{p} )} \right). \\ \end{aligned} $$

Equation (12) is further elaborated in the next section.

It is notable that classical statistical methods, such as the independent samples *t* test and the paired *t* test, rely on strong independence assumptions between measurements. Specifically, the independent samples *t* test assumes that two quantities originating from different biological samples are uncorrelated, i.e., $\text {Cov}(x_{c_1i_1r_1}^p, x_{c_2i_2r_2}^p) = 0$ whenever $c_1 \ne c_2$ or $r_1 \ne r_2$ÿ. Similarly, the paired *t* test assumes that $\text {Cov}(x_{c_1i_1r_1}^p, x_{c_2i_2r_2}^p) = 0$ whenever the two quantities come from different replicates or fragment ions ($i_1 \ne i_2$ or $r_1 \ne r_2$). By contrast, the proposed method does not impose such restrictive assumptions; instead, it estimates the covariance terms directly through the hierarchical graphical model introduced in the previous section in combination with shrinkage estimation.

#### Structure of covariance matrix

To determine which of the covariance terms, $\text {Cov}(x_{c_1i_1r_1}^p, x_{c_2i_2r_2}^p)$, are nonzero, we use the hierarchical graphical model presented in (2) to (7). We note that $x_{cir}^p = \mu ^p + \delta _c + \zeta _c^p + e_{cr}^p + \xi _{cir}^p$, where $\zeta _c^p = \log _{10} u_c^p$ and $\xi _{cir}^p = \log _{10} w_{cir}^p$. It follows that $\text {Cov}(x_{c_1i_1r_1}^p, x_{c_2i_2r_2}^p) = \text {Cov}(\zeta _{c_1}^p, \zeta _{c_2}^p) + \text {Cov}(e_{c_1r_1}^p, e_{c_2r_2}^p) + \text {Cov}(\xi _{c_1i_1r_1}^p, \xi _{c_2i_2r_2}^p),$ while the peptide *p* is fixed. The nonzero covariance terms are determined as described below.

1. In the case that $c_1 = c_2 \equiv c$ and $r_1 = r_2 \equiv r$, then,
  13 $$\begin{aligned} \begin{aligned} a_{i_1i_2}&\equiv \text {Cov}(x_{ci_1r}^p, x_{ci_2r}^p) \\&= \text {Var}(\zeta _{c}^p) + \text {Var}(e_{cr}^p) + \text {Cov}(\xi _{ci_1r}^p, \xi _{ci_2r}^p), \end{aligned} \end{aligned}$$
  for all *c*, $i_1$, $i_2$, and *r*.
2. In the case that $c_1 \ne c_2$, and $r_1 = r_2 \equiv r$, then,
  14 $$\begin{aligned} b_{i_1i_2} \equiv \text {Cov}(x_{c_1i_1r}^p, x_{c_2i_2r}^p) = \text {Cov}(\xi _{c_1i_1r}^p, \xi _{c_2i_2r}^p), \end{aligned}$$
  for all $c_1 \ne c_2$, $i_1$, $i_2$, and *r*.
3. In the case that $c_1 = c_2 \equiv c$ and $r_1 \ne r_2$, then,
  15 $$\begin{aligned} d \equiv \text {Cov}(x_{ci_1r_1}^p, x_{ci_2r_2}^p) = \text {Var}(\zeta _{c}^p), \end{aligned}$$
  for all *c*, $i_1$, $i_2$, and $r_1 \ne r_2$.

**Fig. 3:**
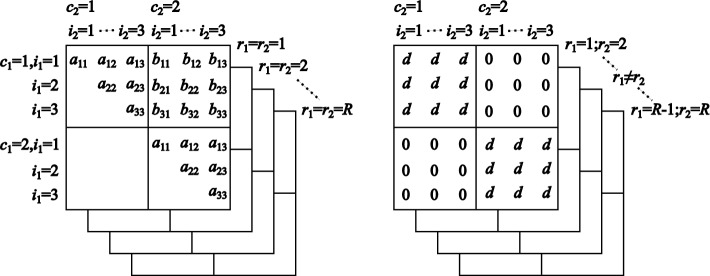
Structure of the covariance matrix comprising the elements $\text {Cov}(x_{c_1i_1r_1}^p, x_{c_2i_2r_2}^p)$. The slices on the left represent cases where $r_1 = r_2$, and those on the right represent cases where $r_1 \ne r_2$. Each slice in the left and right panels takes the form of a $2I \times 2I$ matrix, since there are $C=2$ conditions and *I* fragment ions, while $r_1$ and $r_2$ are fixed

Figure 3 illustrates the structure of the covariance matrix, which consists of the elements $\text {Cov}(x_{c_1i_1r_1}^p, x_{c_2i_2r_2}^p)$ for the proposed hierarchical graphical model. The entries $a_{i_1i_2}$, $b_{i_1i_2}$, and *d* are defined as described above. Notably, the log-transformed fragment ion quantities are correlated even in cases where $c_1 \ne c_2$ and $r_1 = r_2$, due to the entries $b_{i_1i_2}$, and in cases where $r_1 \ne r_2$ and $c_1 = c_2$ due to the entry *d*.

The variance term in (12) can be re-written in a simpler form as follows: assuming that $R_1 = R_2 \equiv R$,

16 $$\begin{aligned} \text {Var}({\hat{L}}_\text {shrink}) = \frac{2}{R} \sum _{i=1}^I \sum _{j=1}^I a_{ij} - \frac{2}{R} \sum _{i=1}^I \sum _{j=1}^I b_{ij} + \frac{2I^2(R-1)}{R} d. \end{aligned}$$

#### Estimation of covariance terms where $r_1 = r_2$

For the estimation of the covariance terms, we separate the case of $r_1 = r_2$ and the case of $r_1 \ne r_2$. To simplify the notation, we will omit the superscript *p* in the following paragraph where it is unlikely to cause confusion.

First, for the cases of $r_1 = r_2$, the corresponding covariance terms, $a_{i_1i_2}$ and $b_{i_1i_2}$, are estimated using the James-Stein-type shrinkage estimation method [16, 17]. We note that $x_{cir}^p$ can be expressed as the sum of its components: $x_{cir}^p = \mu ^p + \delta _c + \zeta _c^p + e_{cr}^p + \xi _{cir}^p$. Based on this representation, the deviation $x_{cir}^p - {\bar{x}}_{ci}^p$ is equivalent to the deviation of $e_{cr}^p + \xi _{cir}^p$ with respect to its sample mean ${\bar{e}}_{c}^p + {\bar{\xi }}_{ci}^p$. Consequently, the sample covariance, $s_{c_1i_1, c_2i_2}$, provides an estimate of the covariance of the components, $\text {Cov}(e_{c_1r}^p + \xi _{c_1i_1r}^p, e_{c_2r}^p + \xi _{c_2i_2r}^p)$, rather than the direct covariance term $\text {Cov}(x_{c_1i_1r}^p, x_{c_2i_2r}^p)$. The $a_{i_1i_2}$ and $b_{i_1i_2}$ in (13) and (14) can be estimated by

17 $$\begin{aligned} \begin{aligned} a_{i_1i_2}&\approx d + s_{ci_1,ci_2}^*,\\ b_{i_1i_2}&\approx s_{c_1i_1,c_2i_2}^*, \end{aligned} \end{aligned}$$

for all $c, c_1, c_2, i_1$, and $i_2$, where $s_{c_1i_1,c_2i_2}^*$ represents a shrinkage estimate of the covariance $\text {Cov}(e_{c_1r}^p + \xi _{c_1i_1r}^p, e_{c_2r}^p + \xi _{c_2i_2r}^p)$. The shrinkage estimate is written in terms of a correlation estimate and variance estimates as:

18 $$\begin{aligned} s_{c_1i_1,c_2i_2}^* = r_{c_1i_1,c_2i_2}^* \sqrt{s_{c_1i_1,c_1i_1}^* s_{c_2i_2,c_2i_2}^*}. \end{aligned}$$

The correlation estimate, $r_{c_1i_1,c_2i_2}^*$, is obtained by shrinking the sample correlation $r_{c_1i_1,c_2i_2}$, computed between the log-transformed fragment ion quantities $x_{c_1i_1r}$ and $x_{c_2i_2r}$, toward zero: $r_{c_1i_1,c_2i_2}^* = (1 - {\hat{\lambda }}) \cdot r_{c_1i_1,c_2i_2},$ if $c_1\ne c_2$ or $i_1 \ne i_2$; $r_{c_1i_1,c_2i_2}^* = 1,$ otherwise, where $0\le {\hat{\lambda }} \le 1$ is the shrinkage intensity parameter [17]. The variance estimate $s_{ci,ci}^*$ is obtained by shrinking the sample variance $s_{ci,ci}$ of $x_{cir}$ toward the median of all sample variances: $s_{ci,ci}^* = (1 - {\hat{\lambda }}_\text {v}) \cdot s_{ci,ci} + {\hat{\lambda }}_\text {v} \cdot s_{\text {median}},$ where $s_{\text {median}}$ denotes the median of $\{ s_{11,11}, s_{12,12}, \ldots , s_{2I,2I} \}$, and $0\le {\hat{\lambda }}_\text {v} \le 1$ is the shrinkage intensity parameter [16].

Note that if the shrinkage parameter ${\hat{\lambda }}$ is set to one, the correlation estimate $r^*_{c_1i_1, c_2i_2}$ is reduced to zero. Setting zero correlation coefficients as the shrinkage target reflects the strong assumption of independence across biological samples and fragment ions—an assumption imposed by classical statistical methods such as the independent samples *t* test and the paired *t* test.

In this paper, the shrinkage parameters $({\hat{\lambda }}, {\hat{\lambda }}_\text {v})$ are computed using the R package corpcor via the function cov.shrink [22]. In corpcor, the optimal values of the shrinkage parameters are obtained by minimizing the mean squared errors (MSEs) in the estimation of correlations and variances, respectively [16, 17].

#### Equal covariance assumption

We assume that the covariances are equal across conditions to reduce the number of parameters to estimate, i.e., $\text {Cov}(x_{1i_1r}^p, x_{1i_2r}^p) = \text {Cov}(x_{2i_1r}^p, x_{2i_2r}^p)$. From the estimated covariances in (18), the pooled covariance estimates can be computed by $s_{\cdot i_1, \cdot i_2}^* \equiv (\cdot s_{1 i_1, 1 i_2}^* + \cdot s_{2 i_1, 2 i_2}^* ) / 2 ,$ for all $i_1$ and $i_2$, which replace $s_{ci_1, ci_2}^*$ in the expression for $a_{i_1i_2}$ in (17).

#### Estimation of covariance terms where $r_1 \ne r_2$

Second, for the cases where $r_1 \ne r_2$ and $c_1 = c_2 \equiv c$, we notice that the covariance terms are equal to the variance, $\text {Var}(\zeta _c^p)$, for all $c, i_1, i_2$, and $r_1 \ne r_2$, as presented in (15). From the hierarchical graphical model described in Sect. 2.1, the $\log _{10}$-transformed peptide quantity is represented as $y_{cr}^p = \mu ^p + \delta _c + \zeta _c^p + e_{cr}^p$. We compute the normalized quantity as $z_{cr}^p = y_{cr}^p - m_c^y + m^y$, where $m_c^y$ represents the median of all $y_{cr}^p$ values under condition *c*, and $m^y$ denotes the median of all $y_{cr}^p$ values across all conditions. This normalization removes variability caused by the condition difference, $\delta _c$. Furthermore, we calculate the sample mean as ${\bar{z}}_c^p = \sum _{r=1}^R z_{cr}^p / R$ for all *c* and *p*. This sample mean can be considered an instance of $\mu ^p + \delta + \zeta _c^p$, where $\delta$ is a fixed constant independent of *c* and *p*. Note that:

19 $$\begin{aligned} \text {Var}(\mu ^p + \delta + \zeta _c^p) = \text {Var}(\mu ^p) + \text {Var}(\zeta _c^p). \end{aligned}$$

The variance, $\text {Var}(\mu ^p + \delta + \zeta _c^p)$, is estimated by calculating the sample variance of all ${\bar{z}}_{c}^p$ values across all conditions and peptides. To estimate the variance of the mean quantity, $\text {Var}(\mu ^p)$, we notice that the empirical distribution for ${\bar{z}}_c^p$ is typically skewed as illustrated in Figure S1(a) of the Additional file 1 in Supplementary Information, whereas the distribution for $\mu ^p$ is symmetric as presented in (8). The variance $\text {Var}(\mu ^p)$ is estimated through the following steps:

1. The mode $M^z$ of the empirical distribution of the ${\bar{z}}_c^p$ values is computed using the kernel density estimation method, implemented in the R package stats using the function density.
2. The ${\bar{z}}_{c}^p$ values are then split into two parts, $Z_1 = \{M^z - {\bar{z}}_c^p \,|\, {\bar{z}}_c^p \le M^z\}$ and $Z_2 = \{{\bar{z}}_c^p - M^z \,|\, {\bar{z}}_c^p > M^z\}$.
3. An estimate of the standard deviation, $(\text {Var}(\mu ^p))^{1/2}$, is computed using a quantile from one of the split parts, divided by the corresponding standard normal quantile. Specifically,
  20 $$\begin{aligned} \left( \widehat{\text {Var}}(\mu ^p) \right) ^{\frac{1}{2}} = \min _{s \in \{1,2\},{\tilde{p}} \ge 0.3} \left\{ \frac{\text {quantile} (Z_s, {\tilde{p}})}{\Phi ^{-1}(0.5 + {\tilde{p}}/2)} \right\} , \end{aligned}$$
  where $\Phi (z) = P(Z\le z)$ is the cumulative distribution function of a standard normal random variable *Z*.

Finally, an estimate of $\text {Var}(\zeta _c^p)$ is computed by subtracting the estimated variances as

21 $$\begin{aligned} d \approx \widehat{\text {Var}}(\zeta _c^p) = \widehat{\text {Var}}(\mu ^p + \delta + \zeta _c^p) - \widehat{\text {Var}}(\mu ^p). \end{aligned}$$

### Bootstrap estimation of degrees-of-freedom

The degrees-of-freedom for the proposed test statistic, $t_\text {shrink}$, are determined using a bootstrap technique known as the additive method [18]. Let ${\mathcal {X}}$ denote the set of independent and identically distributed random variables ${\mathcal {X}} = \{z_r^p\}, r=1,\ldots ,R,$ with $z_r^p = (x_{11r}^p, \ldots , x_{1Ir}^p, x_{21r}^p, \ldots , x_{2Ir}^p)$ representing the list of fragment ion quantities for peptide *p* in the *r*th replicate. Let ${\mathcal {X}}^*_1, \ldots , {\mathcal {X}}^*_B$ be *B* random samples of size *R*, generated by independently sampling elements from ${\mathcal {X}}$ with replacement. For each sample ${\mathcal {X}}^*_b$, bootstrap shrinkage-based test statistics are computed as:

22 $$\begin{aligned} t_{\text {shrink},b}^* = \frac{{\hat{L}}_{\text {shrink},b}^*}{\text {SE}({\hat{L}}_{\text {shrink},b}^*) + a_R}, \end{aligned}$$

where $a_R > 0$ is a fixed constant that prevents the denominator from approaching zero [18]. We remark that the number of replicates *R* is often as small as $R=4$, in which case the standard error term in the denominator may degenerate to zero because of duplicates in bootstrap samples, leading to unstable values. The value of $a_R$ is determined at the minimum point of the coefficient of variation (see Sect. 3.1.1). Let $s^{*2}$ denote the sample variance of the *B* test statistics, $t_{\text {shrink},1}^*,\ldots ,t_{\text {shrink},B}^*$. The degrees-of-freedom are determined based on the relationship between the variance and the degrees-of-freedom of Student’s *t* distribution:

23 $$\begin{aligned} \nu _\text {shrink} = \frac{ 2 s^{*2} }{ s^{*2} - 1 } \end{aligned}$$

for $s^{*2} > 1$, and $\nu _\text {shrink} = \infty$ for $s^{*2} \le 1$.

## Results

The proposed method was compared with two classical statistical methods–paired *t* test and independent samples *t* test–and two modern statistical methods–ROTS [14] and MSstatsLiP [19]–using simulated data sets and real MS data sets. Among the compared methods, the independent samples *t* test and ROTS are two-step methods, whereas the paired *t* test and MSstatsLiP are feature-based methods. Notably, the proposed method is designed for differential analysis of DIA-MS data at the precursor peptide-level, rather than at the protein-level. Other widely used statistical methods such as MSqRob and MSstats were not included in the comparison, as they are designed for protein-level analysis. The limma [23] will be integrated and evaluated for future work. All statistical methods numerically evaluated in this study are conveniently accessible via the R package MDstatsDIAMS (https://github.com/namgillee/MDstatsDIAMS).

### Simulation experiments

Simulated data sets were generated using the hierarchical graphical model described in Sect. 2.1 to compare the performance of the proposed shrinkage-based method with two other classical statistical methods. The model parameters were chosen to ensure that the generated data distributions closely match the sampling distributions presented in Sect. S2 of the Additional file 1 in Supplementary Information. By default, the number of fragment ions was set to $I=3$, the number of peptides to $P=500$, and the number of replicates to $R=4$. Default values for hyperparameters were set as $\sigma _c^p = 0.2$ for (3), $\alpha _0^p = 2$ and $\beta _0^p = 10$ for a beta distribution in (6), $(\alpha _1^p,\alpha _2^p,\alpha _3^p) = (2, 2, 2)$ for (7), and $\mu = 5.0$ and $\sigma = 1.0$ for (8). The correlation, $\rho ^p$, between $w_{1ir}^p$ and $w_{2ir}^p$ was set to 0.89 by default unless otherwise specified.

For the evaluation of hypothesis testing methods, we generated simulated data sets under two conditions with $\mu _1^p = \mu ^p$ and $\mu _2^p = \mu _1^p + \delta$, where $\delta \ge 0$ was varied as $\delta = 0$, $\log _{10} (2) \approx 0.3$, and $\log _{10} (4) \approx 0.6$. After evaluating the results on *P* peptides, we computed the specificity for $\delta =0$ and the sensitivity for $\delta > 0$. The accuracy was then calculated based on a contingency table, which included 95% of the cases with $\delta = 0.0$ and 5% with a specific value of $\delta > 0$.

#### Estimation of degrees-of-freedom

To determine an appropriate value for $a_R$ in (22), we generated $B=100$ bootstrap samples under the conditions $\mu _1^p = \mu ^p$ and $\mu _2^p = \mu _1^p + \delta$ with $\delta =0, 0.3, 0.6$, and calculated the coefficient of variation (CV) for the bootstrap shrinkage-based test statistics $t_{\text {shrink},b}^*$, where $b=1,\ldots ,B$. Figure S6 of the Additional file 1 in Supplementary Information displays the CV values across different $a_R$ values. The figure reveals that the curve is nearly flat, while fluctuation is relatively large for small $a_R$ values. We selected $a_R$ as $a_4 = 0.3$ for $R=4$ based on Figure S6 of the Additional file 1 in Supplementary Information, where the change in the CV is less than 10% of its maximum value. To ensure the asymptotic consistency of the proposed test statistics for larger *R* values, we set $a_R$ as $a_R = a_4 \times (R / 4)^{-3/2} = 2.4 R^{-3/2}$ [18].

#### Consistency of estimation

**Fig. 4:**
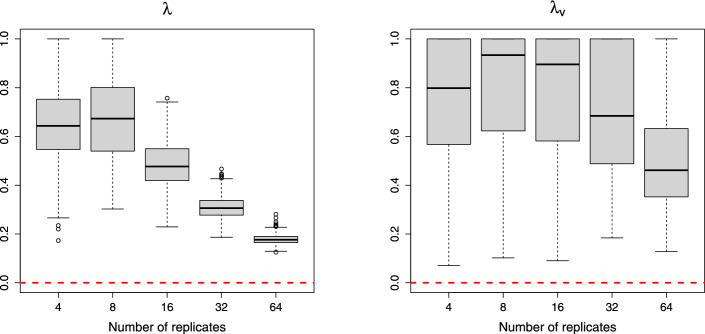
Convergence of the estimated shrinkage intensity parameter values, ${\hat{\lambda }}$ (left) and ${\hat{\lambda }}_\text {v}$ (right). The red dotted line indicates the zero value

The proposed method estimates the covariance terms, $\text {Cov}(x_{c_1i_1r_1}^p, x_{c_2i_2r_2}^p)$, as analyzed in Sect. 2.2.1 Using simulated data sets generated with the default parameter values, equal mean quantities $\mu _1^p = \mu _2^p = 5.0$, and increasing numbers of replicates ($R=4$ to $R=64$), we compared the estimated values of $a_{ij}, b_{ij},$ and *d* with their true theoretical values. Fig. 4 shows the estimated shrinkage intensity parameter values, ${\hat{\lambda }}$ and ${\hat{\lambda }}_\text {v}$. The results clearly indicate that the shrinkage intensities decrease toward zero as the number of replicates increases.

**Fig. 5:**
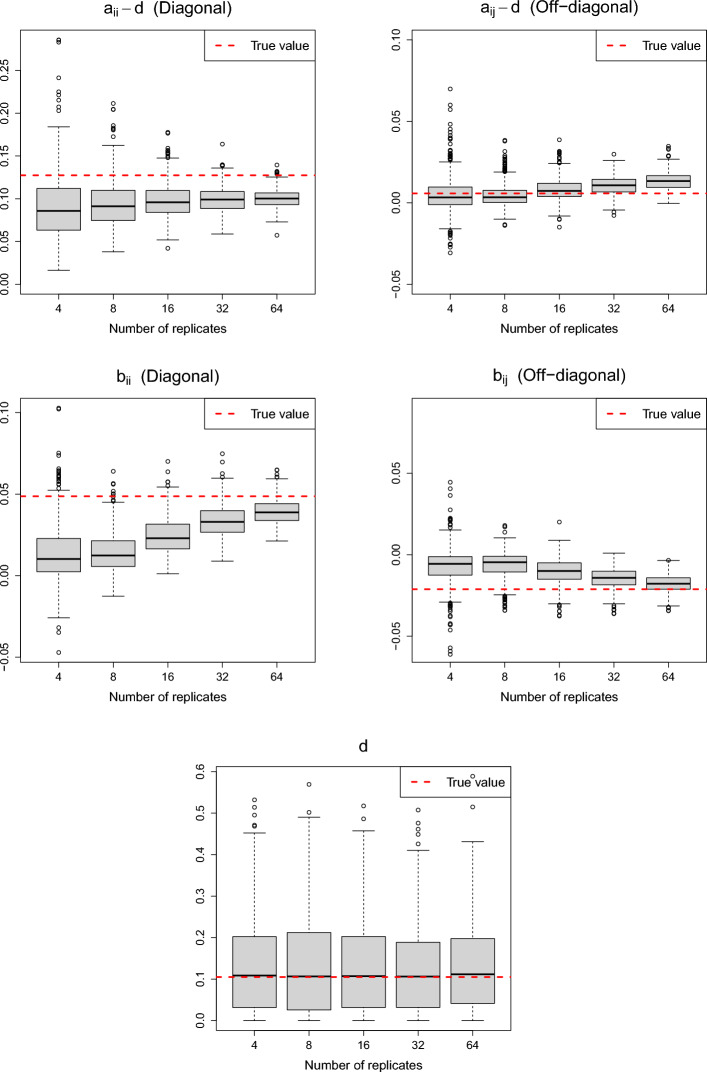
Convergence of the estimated covariance terms: $a_{ii} - d$ (top left), $a_{ij} - d$ with $i\ne j$ (top right), $b_{ii}$ (middle left), $b_{ij}$ with $i \ne j$ (middle right), and *d* (bottom). The red dotted line indicates the true values corresponding to the estimates

Figure 5 compares the estimated covariance terms with their corresponding true values as the number of replicates increases. From the theoretical results in Proposition S1 of the Additional file 1 in Supplementary Information, we can derive that $\text {Var}(\zeta _c^p) = (\psi '(2) - \psi '(12)) / (\log (10))^2 = 0.1053,$ $\text {Var}(e_{cr}^p) = (\sigma _c^p)^2 = 0.04,$ $\text {Var}(\xi _{cir}^p) = (\psi '(2) - \psi '(6)) / (\log (10))^2 = 0.0874.$ Additionally, the covariances $\text {Cov}(\xi _{c_1i_1r}^p, \xi _{c_2i_2r}^p)$, involved in (13) and (14), were calculated using Monte Carlo simulations with Dirichlet distributions. Using these results, the true values for the covariance terms based on (13) to (15) are $a_{ii} - d = 0.04 + 0.0874 = 0.1274$, $a_{i_1i_2} - d = 0.04 - 0.0342 = 0.0058$ for $i_1 \ne i_2$, $b_{ii} = 0.0487$, $b_{i_1i_2} = -0.0212$ for $i_1 \ne i_2$, and $d = 0.1053$.

The top panels of Fig. 5 show that the estimates of $a_{ii} - d$ and $a_{ij} - d$ ($i\ne j$) approach the true values as the number of replicates increases. Specifically, the variances of the estimates decrease, with small biases, leading to more accurate estimations. The middle panels illustrate that the estimates of $b_{ii}$ and $b_{ij}$ $(i\ne j)$ converge toward their true values as the number of replicates increases. Finally, the bottom panel demonstrates that the estimate for *d* is unbiased, although it shows relatively high variance.

Figure S7 of the Additional file 1 in Supplementary Information shows the empirical distribution of the shrinkage-based test statistic for cases with $R=4$ and $R=64$ replicates. In both cases, $P=500$ test statistics and degrees-of-freedom were calculated, and the median of the calculated degrees-of-freedom was $\infty$ in both cases. The figure demonstrates that the empirical distribution of the test statistic closely aligns with the *t*-distribution.

#### Numerical evaluation

To compare the performance of the proposed shrinkage-based method with the four other classical methods, simulated data sets were generated under various model parameters including the number of replicates $R=4,8,\ldots ,64$, the hyperparameters for the Beta distribution $(\alpha _0^p, \beta _0^p) = (2,10), (4, 28), (11, 91)$, and the noise standard deviation $\sigma _c^p = 0.05, 0.1,\ldots , 0.8$, while the rest of the parameters remained fixed.

##### Specificit

**Fig. 6:**
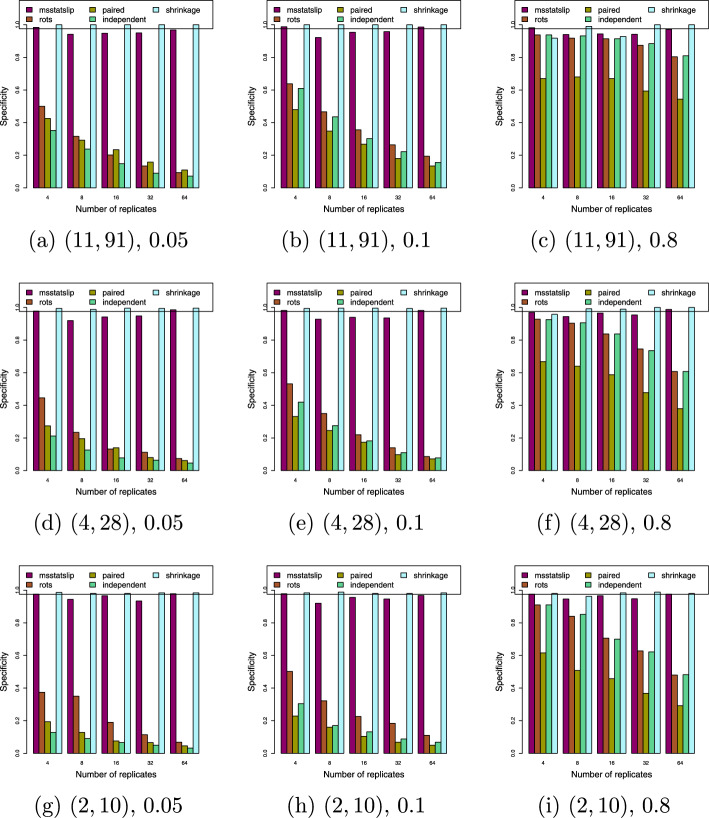
Specificity obtained using the five statistical testing methods: the MSstatsLiP, ROTS, paired *t* test, independent samples *t* test, and shrinkage *t* test. The model parameters $(\alpha _0^p, \beta _0^p)$ and $\sigma _c^p$ are specified below each figure panel

 Using a significance level of $\alpha =0.05$, specificity was calculated as the proportion of correctly classified peptides among those satisfying the true null hypothesis $H_0: \mu _1^p = \mu _2^p$. Fig. 6 illustrates the specificities produced by the five statistical methods under various model parameters. In cases of small noise standard deviations ($\sigma _c^p = 0.05, 0.1$), the shrinkage method achieved the highest specificities in most cases. For large noise standard deviations ($\sigma _c^p = 0.8$), the MSstatsLiP yielded the highest specificities for small *R* values, while the shrinkage method performed well for large *R* values.

In contrast, the paired *t* test consistently produced lower specificities under medium and high noise standard deviations. This result suggests that the paired *t* test rejects null hypotheses more frequently than the other methods due to the underestimation of standard errors and the overestimation of degrees-of-freedom. On the other hand, the shrinkage *t* test, which is also a feature-based method like the paired *t* test, improves performance by employing shrinkage-based covariance estimation to more accurately compute the standard errors used in the test statistic.

##### Sensitivity

 Sensitivity was calculated as the proportion of correctly classified peptides among those with different mean log-quantities between conditions ($\mu _1^p \ne \mu _2^p$). Figure S8 of the Additional file 1 in Supplementary Information illustrates the sensitivities produced by the five statistical methods when the mean difference was set to $\delta = \log _{10}(2)$. In the figure, the paired *t* test consistently achieved higher sensitivity values compared to the other methods. This result is due to its tendency to reject the null hypothesis more frequently, as verified by its low specificities observed in the previous section. Although the other methods exhibited lower sensitivities than the paired *t* test, the performance reduction was minimal in cases with small noise standard deviations ($\sigma _c^p = 0.05, 0.1$), which better reflect the characteristics of real MS data distributions, as shown in Figure S1(b) of the Additional file 1 in Supplementary Information.

##### Accuracy

 Accuracy was calculated as the proportion of correctly classified peptides from a mixture of those satisfying the null hypothesis and those not. For this calculation, we assumed that 95% of the peptides satisfied the null hypothesis, while the remaining 5% did not. The proportion reflects the realistic scenario where the number of drug targets showing drug-induced modulation in peptide quantities is much smaller than the number of non-targets.

Figure S9 of the Additional file 1 in Supplementary Information shows the accuracy of the five statistical methods in cases where the mean log-quantities of peptides differed between conditions by $\delta = \log _{10}(2)$. The figure demonstrates that the shrinkage *t* test achieved the highest accuracy among the five methods in almost all cases. This result suggests that the shrinkage *t* test performs best when the data distribution closely resembles real MS data distributions, while maintaining low false positives, as indicated by the high specificities shown in Fig. 6.

### Mass spectrometry data analysis

The original MS raw files are publicly available through the ProteomeXchange Consortium via the PRIDE repository, with the dataset identifier PXD015446 [24]. We selected raw files from HeLa cell lysates treated with the kinase inhibitor Staurosporine, along with control samples processed under similar conditions. The DIA raw files were analyzed using Spectronaut 17, MaxQuant 2.4.10, and Skyline 25.1 with their respective default settings, except that the digest type was set to semi-specific. For further details on raw MS data processing, see Sect. S1 of the Additional file 1 in Supplementary Information.

The real MS datasets were further analyzed using five statistical methods: the MSstatsLiP, ROTS, paired *t* test, independent samples *t* test, and shrinkage *t* test. Each peptide was tested for differences in the mean log-quantities between two conditions, dimethyl sulfoxide (DMSO) and the drug-treatment condition, where the drug concentration varied from 100 pM to 100 µM.

The shrinkage *t* test method estimated the parameter $d = \text {Var}(\zeta _{c}^p)$ for each comparison of two conditions, as described in Sect. 2.2.4. Table S2 of the Additional file 1 in Supplementary Information summarizes the estimated values of *d*. The table shows that the estimates are consistent across different comparisons, likely due to the large number of peptides involved in each comparison. This consistency indicates that the estimated values are reliable for real data analysis.

Table 2 summarizes sensitivities of the five statistical methods at different specificity levels. The methods tested differences in mean log-quantities between the control vehicle (DMSO) and the drug-treatment condition at 100 µM, because proteins were labeled as positive if their binding affinity (dissociation constant, Kd) was reported to be lass than 10 µM in the literature [25], and as negative otherwise. After computing the * p* values for comparing two conditions using each statistical method, we applied the local false discovery rate (lfdr) approach [26] to calculate fdr scores for multiple hypothesis testing. Then, we assigned the minimum fdr score among the peptides originating from a given protein as the fdr score for that protein [27]. The fdr scores aggregated at the protein level were used to determine statistical significance. The table shows that the proposed shrinkage *t* test method achieved the highest sensitivity values of 0.69 and 0.84 at specificity levels of 0.4 and 0.2, respectively, indicating its enhanced effectiveness in detecting peptides with changes in mean log-quantities.

**Table 2:** Sensitivities obtained using the five statistical methods were evaluated for different specificity values in testing differences in mean log-quantities between the control vehicle (DMSO) and the drug-treatment condition at 100 µM

Sensitivty	Specificity			
*Spectronaut*	0.8	0.6	0.4	0.2
MSstatsLiP	**0**.**26**	**0**.**44**	0.63	0.81
ROTS	**0**.**26**	0.42	0.60	0.80
Paired	0.19	0.41	0.60	0.80
Independent	**0**.**26**	**0**.**44**	0.63	0.81
Shrinkage	0.20	0.41	**0**.**69**	**0**.**84**
*MaxQuant*	0.8	0.6	0.4	0.2
MSstatsLiP	0.21	0.38	0.57	0.78
ROTS	0.20	0.40	0.59	0.80
Paired	0.19	0.40	0.60	0.80
Independent	0.20	0.40	0.61	0.80
Shrinkage	0.20	0.40	0.62	0.81
*Skyline*	0.8	0.6	0.4	0.2
MSstatsLiP	0.20	0.40	0.60	0.80
ROTS	0.20	0.41	0.60	0.80
Paired	0.14	0.36	0.58	0.79
Independent	0.20	0.40	0.60	0.80
Shrinkage	0.20	0.40	0.60	0.80

Boldface emphasizes the highest sensitivity value across all five methods and three software platforms

On the other hand, pairwise comparison results between every pair of drug conditions were aggregated for each peptide to evaluate its significance in the dose-response relationship. The statistical significance of a peptide’s dose-response relationship was determined by combining * p* values from consecutive pairwise comparisons, DMSO/10 nM, 10 nM/100 nM, and 100 nM/100 µM, as follows:

24 $$\begin{aligned} {\tilde{p}}_\text {drc} = \prod _{i: s_i = s_m} {\tilde{p}}_i \prod _{j: s_j \ne s_m} (1 - 0.5 {\tilde{p}}_j), \end{aligned}$$

where ${\tilde{p}}_i$ is the p-value of the *i*-th comparison, ${\tilde{p}}_m$ is the minimum p-value among the comparisons, and $s_i$ and $s_m$ are the signs of the estimates corresponding to ${\tilde{p}}_i$ and ${\tilde{p}}_m$, respectively. The significance value, ${\tilde{p}}_\text {drc}$, was further summarized at the protein level by taking the minimum value across peptides for each protein. The area under the receiver operating characteristic curve (AUC) was then evaluated based on the significance of each protein. Table 3 presents the AUCs for each method, showing that the proposed shrinkage method achieved the highest AUC score.

**Table 3:** AUC values obtained using the five statistical methods. The AUCs were computed based on the statistical significance described in (24), derived from a sequence of consecutive pairwise comparisons: DMSO/10 nM, 10 nM/100 nM, 100 nM/100 µM

AUC values	Spectronaut	MaxQuant	Skyline
MSstatsLiP	0.489	0.545	0.502
ROTS	0.539	0.506	0.500
Paired	0.532	0.551	0.511
Independent	0.531	0.557	0.516
Shrinkage	0.537	**0**.**599**	0.521

Boldface emphasizes the highest AUC value across all five methods and three software platforms

We further investigated the analysis results of the proposed method using the Spectronaut report. Since the results are peptide-specific, it is possible to detect precursor peptides exhibiting significant quantitative changes between drug treatment conditions. These peptides can be used to infer the location of drug-binding pockets on target proteins, as illustrated below.

**Fig. 7:**
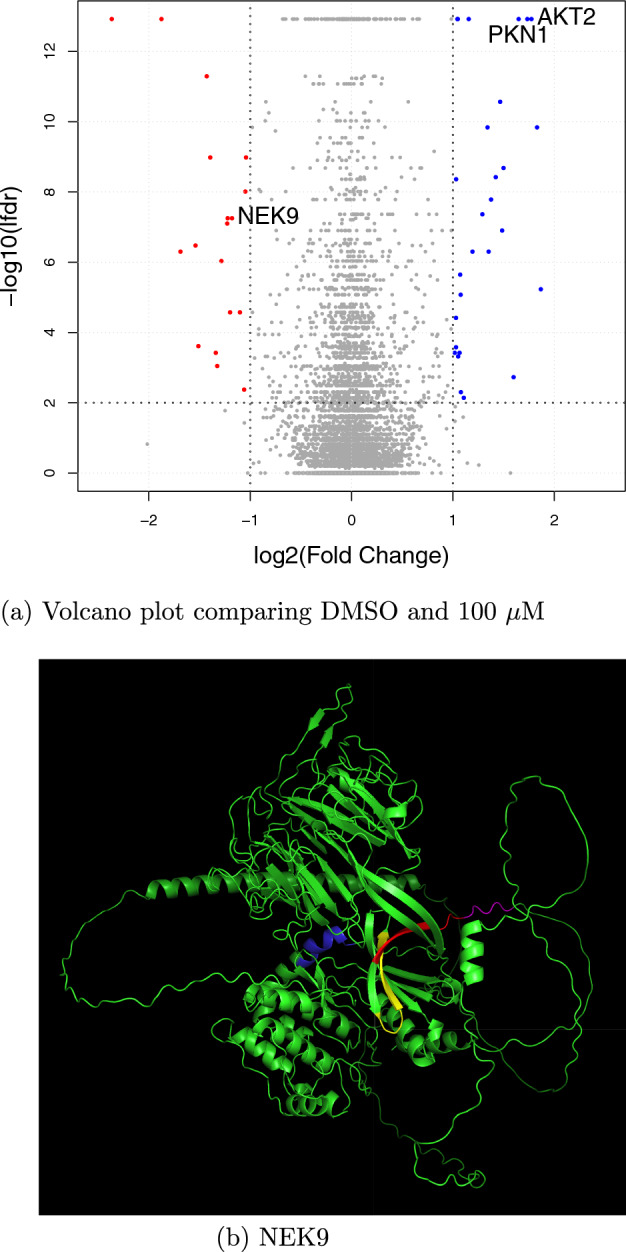
**a** Volcano plot comparing the DMSO and 100 $\mu$M conditions. The lfdr scores were aggregated at the protein-level. Three kinase proteins, NEK9, AKT2, and PKN1, are selected and illustrated in the figure. **b** 3D structure of the kinase protein NEK9, with the four detected peptides highlighted in colors. The 3D structure was obtained from AlphaFold (https://alphafold.ebi.ac.uk/entry/Q8TD19)

Figure 7a shows a protein-level volcano plot comparing the DMSO and 100 $\mu$M conditions. The vertical axis represents the negative $\hbox {log}_{10}$-transformed local false discovery rate (lfdr) scores, aggregated at the protein-level, and the horizontal axis shows the $\hbox {log}_2$ fold change. The lfdr score allows for effective control of false positive rates. Based on the volcano plot, we selected three target kinase proteins that satisfied the criteria of lfdr $\le 0.01$ and $| \log _2(\text {fold change}) | \ge 1.$ For each selected protein, we identified peptides with significant changes in abundance. Fig. 7b visualizes the 3D structure of the kinase protein NEK9. Four peptides with significant quantitative changes were detected: AEQEELHYIPIR, AGGGAAEQEELHYIPIR, GAFGEATLYR, and VTLLNAPTK. These peptides are highlighted in red, magenta, yellow, and blue, respectively. Based on their spatial positions in the 3D structure, it is possible to approximately localize the drug-binding pocket.

These structural analysis results highlight the utility of peptide-level differential expression analysis. Additional 3D structures of the other two kinase proteins, AKT2 and PKN1, with the detected peptides highlighted in red, are shown in Figure S10 in Additional file 1 of the Supplementary Information.

## Discussion and conclusions

In quantitative bottom-up proteomics, the instrument’s quantification performance depends on the loss or detection efficiency of the measured ions [28]. The total ion loss and its variability significantly affect peptide identification, and quantification performance, and throughput. In this study, we introduced a hierarchical graphical model for simulating tandem MS data in quantitative bottom-up proteomics using DIA-MS, in which ionization efficiency and data acquisition rate are incorporated as latent variables. Our results suggest that variations in ionization efficiency and data acquisition rates affect the performance of statistical methods. To address this, we developed a new statistical method using a shrinkage approach that incorporates these variations into the analysis, resulting in a more noise-robust and accurate test statistic. Simulated experiments and real data analyses demonstrated the effectiveness of the proposed method in handling hierarchically generated data with small sample sizes and biases.

This study proposes a statistical method for differential downstream analysis at the peptide-level. In contrast, traditional downstream analyses have mostly focused on protein-level inference, such as MSstats [3]. More recently, peptide-centric approaches have gained increasing attention, particularly in chemical proteomics, where the goal is to identify specific binding sites for drug candidates. For example, the MSstatsLiP [19] extends MSstats to investigate peptide-level quantitative changes in limited proteolysis mass spectrometry (LiP-MS) data. Other recent software tools for peptide-level chemical proteomics include LiPAnalyzeR [29], Flippr [30], and PELSA-Decipher [31].

For future work, the proposed hierarchical graphical model could be extended to incorporate strategies for handling missing data. Missing value imputation may be applied as a preprocessing step, as in MSstats, or embedded within the model itself, as in Triqler [32]. Triqler, a hierarchical Bayesian framework for protein-level differential analysis, offers the advantages of Bayesian approaches, which simplify model parameter estimation while improving robustness to noise and limited sample sizes. Alternatively, non-Bayesian approaches have also emerged. For example, the tree-based quantification method AlphaQuant [33] addresses missing values using counting statistics rather than direct imputation. These developments highlight the need for integrated methodologies that jointly address quantification and missing value handling within the hierarchical structure of bottom-up proteomics data.

## Additional file

Additional file 1

## Data Availability

We used the datasets deposited by other researcher and available at the PRIDE repository, with the dataset identifier PXD015446, https://proteomecentral.proteomexchange.org/cgi/GetDataset?ID=PXD015446.

## Abbreviations

ANOVA: Analysis of variance
AUC: Area under the receiver operating characteristic curve
CV: Coefficient of variation
DDA: Data-dependent acquisition
DIA: Data-independent acquisition
DMSO: Dimethyl sulfoxide
Kd: Dissociation constant
LC-MS/MS: Liquid chromatography coupled with tandem mass spectrometry
lfdr: Local false discovery rate
MS: Mass spectrometry
SWATH-MS: Sequential window acquisition of all theoretical mass spectra
