# Supplementary material for: A shrinkage-based statistical method for testing group mean differences in quantitative bottom-up proteomics
Source: BMC Bioinformatics. 2025 Oct 31;26:269. doi: 10.1186/s12859-025-06275-1 (PMC12577184; doi:10.1186/s12859-025-06275-1)
Supplement: Supplementary file 1 — Additional file 1 [file 12859_2025_6275_MOESM1_ESM.pdf]

Supplementary Materials for the Manuscript: A Shrinkage-based Statistical Method for Testing Group Mean Differences in Quantitative Bottom-up Proteomics

Namgil Lee Hojin Yoo Juhyoung Kim Heejung Yang*

S1 Real MS Data Processing

The original MS raw files are publicly available through the ProteomeXchange Consortium via the PRIDE repository, with the dataset identifier PXD015446 (Piazza et al., 2020). We selected raw files from HeLa cell lysates treated with the kinase inhibitor Staurosporine and control samples processed under similar conditions. The raw files were acquired using either DDA or DIA modes on a Q-Exactive HF-X mass spectrometer (Thermo Scientific). The DDA raw files were acquired from 10 sample fractions, with file names formatted as "E_D180807_S480_Exp36-StauroDoseResp-SpecLib-*_MSG_R01_T0.raw". The DIA raw files were acquired from 32 MS runs, corresponding to $R = 4$ replicates and $C = 8$ conditions, with file names formatted as "E_D180810_S480-#36-StauroDoseResp-*_MHRM_R01_T0.raw". The eight conditions represent varying concentrations of Staurosporine, including one control vehicle.

The DDA and DIA raw files were analyzed by Spectronaut 17, MaxQuant 2.4.10, and Skyline 25.1, with the UniProt FASTA database of human proteomes (20,360 protein entries, version July 2018):

- Spectronaut: The DDA raw files were searched using Biognosys' search engine, Pulsar, in Spectronaut 17 with default search settings except that the digest type was set to semi-specific. The resulting search archive was then used to generate a spectral library consisting of 7,892 protein groups, 152,369 stripped peptides, 190,172 precursors, and 2,544,667 fragment ions. The DIA raw files were also analyzed using Spectronaut 17, using the default settings but with the digest type set to semi-specific.

*heejyang@kangwon.ac.kr

- MaxQuant: The DDA raw files were analyzed in the Standard mode, with the digestion type parameter set to semi-specific. The DIA raw files were analyzed by MaxDIA using the DDA analysis results and the digestion type parameter set to semi-specific and the match-between-runs parameter on.
- Skyline: The spectral library generated by Spectronaut was imported, and the DIA raw files were analyzed with the digestion type option set to semi-specific.

The produced DIA report files are publicly available at
https://zenodo.org/records/15653980.

Table S1: Numbers of identified protein groups, precursors, and fragment ions from the real DIA-MS data analyzed with Spectronaut 17. For sample preparation, the HeLa cells were processed under the limited-proteolysis (LiP) protocol and treated with Staurosporine in multiple concentrations. The mean and standard deviation of $\log_{10}$-transformed precursor quantity were computed for each drug concentration.

Condition	DMSO	100 pM	1 nM	10 nM	100 nM	1 µM	10 µM	100 µM
# Proteins	6,877	6,917	6,912	6,912	6,922	6,894	6,905	6,945
# Stripped peptides	129,959	130,165	130,490	130,021	130,291	129,278	128,991	131,297
# Modified peptides	137,170	136,983	137,572	137,044	137,249	136,108	135,749	138,380
# Precursors	162,350	162,488	163,002	162,643	162,838	161,447	160,969	164,097
# Fragment ions	506,300	510,543	512,074	510,982	512,25	507,241	504,715	516,455
Mean $\log_{10}$ Quantity	4.43	4.44	4.44	4.44	4.44	4.43	4.44	4.42
SD $\log_{10}$ Quantity	0.74	0.73	0.73	0.73	0.73	0.73	0.74	0.73

S2 Real MS Data Exploration

We investigated the distributions underlying real MS data in detail from the perspective of the hierarchical graphical model proposed in Section Methods. In particular, we demonstrate that the distributions of peptide and fragment ion quantities from real MS experiments are well explained by the proposed model.

Table S1 summarizes the characteristics of the real DIA-MS data set used for the analysis.

Figure S1 illustrates the distribution of precursor quantities from real MS experiments corresponding to a single condition ("DMSO") and four replicates. Specifically, Figure S1(a) shows the distribution of the sample mean of $\log_{10}$-transformed precursor quantities, where the average is computed over the four replicates for each precursor. It is clear that sampling distribution for the mean, $\tilde{\mu}_c^p$, is bell-shaped but skewed. Figure S1(b) shows the histogram of the deviance of $\log_{10}$-transformed precursor quantities, i.e., the transformed quantities were subtracted by the average of the four replicates for each precursor. The figure indicates that the distribution is symmetric, but it has a sharper peak and heavier tails than normal distributions.

Each distribution illustrated in Figure S1 can be explained as a mixture of certain base distributions, as it represents the distribution for all quantified precursors in the experiments rather than a single precursor. In Equation (2), a peptide quantity is represented as a sum of a mean component and an error term. Regarding the error term, it has been known in the literature that heavy-tailed distributions, such as Student's t-distribution and Laplace distribution, can be expressed as mixtures of normal distributions (West, 1987). It implies that the normal distribution assumption for the error term in Equation (2) is appropriate for modeling the empirical distribution of deviations shown in Figure S1(b).

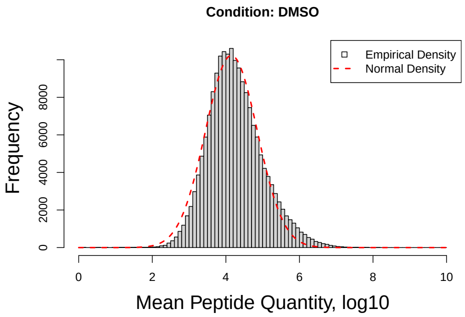

(a)

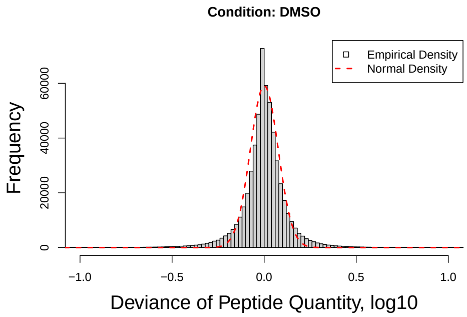

(b)

Figure S1: Distribution of precursor quantities from real MS experiments corresponding to a single condition ("DMSO") and four replicates. (a) Histogram of the sample mean of $\log_{10}$-transformed peptide quantities, and (b) histogram of the deviance of $\log_{10}$-transformed peptide quantities. The red dotted lines indicate normal density functions fitted to the empirical distributions.

On the other hand, to address the skewness of the empirical distribution in Figure S1(a), the proposed hierarchical graphical model allows for biases in the mean of precursor quantities, as expressed in Equation (3), where the mean component is modeled as a sum of a normal random variable, μ_c^p, and the logarithm of a beta random variable, $\log_{10} u_c^p$. Figure S2(a) displays the normal quantile plot for the mean of $\log_{10}$-transformed peptide quantities. Figure S2(b) illustrates the distribution of the simulated data acquisition rates, u_c^p, while Figure S2(c) presents the normal quantile plot of the simulated means of $\log_{10}$-transformed peptide quantities, $\tilde{\mu}_c^p$. Additional details can be found in the caption of Figure S2. From the figure, we observe that the skewness in the empirical distribution of mean precursor quantities may result from the bias introduced by data acquisition rates. Specifically, the heavy left tail of the distribution arises from the logarithmic transformation of the beta random variable, while the heavy right tail is attributed to the right-skewed distribution of data acquisition rates shown in Figure S2(b).

Ionization efficiency, w_{cir}^p, can be directly calculated from data by using the definition in Equation (1). Precursor quantity is estimated by summing peak areas of the top-I fragment ions, where $I = 1, 2, \ldots, 6$. Figure S3 shows histograms of w_{cir}^p for $I = 1, 2, \ldots, 6$ fragment ions from real MS experiments under a single condition ("DMSO") across four replicates. The red dotted lines represent beta distributions fitted to the empirical distributions using maximum likelihood estimation. The figure indicates that ionization efficiency approximately follows beta distributions, which are

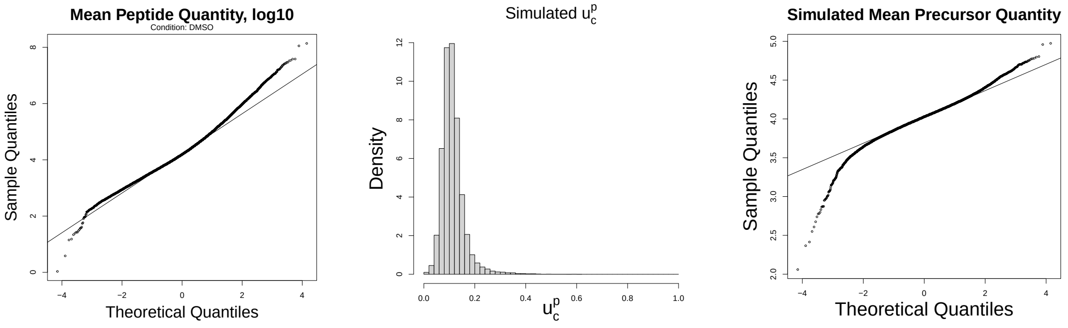

(a) Sample mean (b) Simulated u_c^p (c) Simulated mean, $\tilde{\mu}_c^p$

Figure S2: (a) Normal quantile plot for the mean of $\log_{10}$-transformed peptide quantities from real MS experiments under a single condition ("DMSO"). (b) The distribution of the 30,000 simulated data acquisition rates, u_c^p, and (c) the normal quantile plot of the 30,000 simulated means of $\log_{10}$-transformed peptide quantities, $\tilde{\mu}_c^p$. Specifically, for (b) and (c), we generated $P = 30,000$ values of μ^p from a normal distribution with a mean of $\mu = 5.0$ and a standard deviation of $\sigma = 0.1$, and $P = 30,000$ values of u_c^p from a mixture of beta distributions with shape parameters (α_0^p, β_0^p), where $\beta_0^p = 9(\alpha_0^p - 1) + 1$ and $\alpha_0^p = 2, 3, \ldots, 21$. The shape parameters determined in this way ensure that each beta distribution has its mode at 0.1, as the mode of a beta distribution is given by $(\alpha_0^p - 1)/(\alpha_0^p + \beta_0^p - 2)$. The values of $\tilde{\mu}_c^p$ are then computed as $\tilde{\mu}_c^p = \mu^p + \log_{10} u_c^p$.

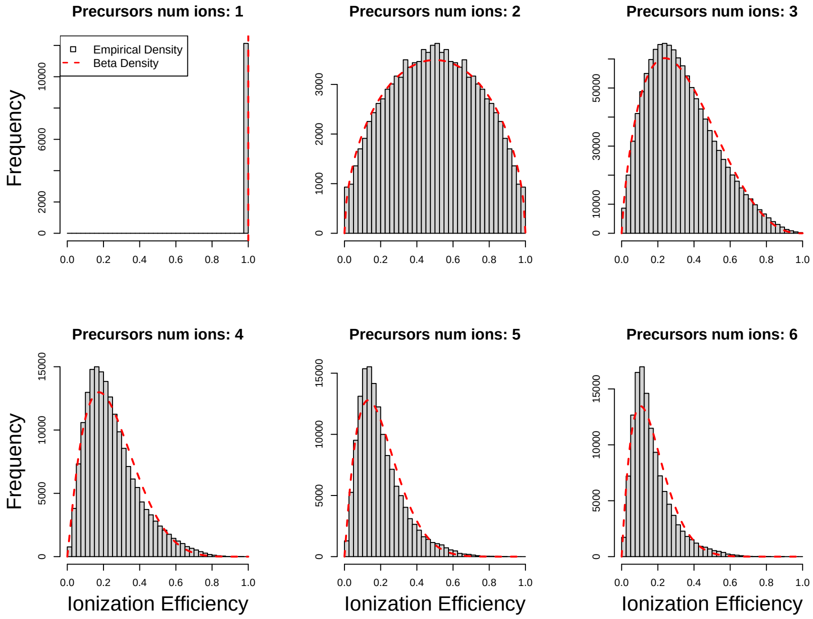

Figure S3: Distributions of ionization efficiency for fragment ion numbers $I = 1, 2, \ldots, 6$, based on real MS data obtained under a single condition ("DMSO") with four replicates. The red dotted lines indicate beta distributions fitted using maximum likelihood estimation.

marginal distributions of Dirichlet distributions.

It is notable that the peak of each distribution shifts toward zero as I increases in Figure S3. To further investigate the distributions of ionization efficiency, we randomly selected 30 proteins, each with at least 30 corresponding precursors, and fitted a beta distribution to the ionization efficiencies derived from precursors with I fragment ions in each protein. Figure S4 presents scatter plot of the estimated shape parameters, α and β, of the beta distributions, categorized by the number of fragment ions, $I = 2, 3, \ldots, 6$. The red dotted lines represent the straight line, $\beta = (I - 1)\alpha$, with a slope of $(I - 1)$ and an intercept of zero. The figure demonstrates that the estimated parameters align perfectly with the straight line, indicating that the mean of the beta distribution is $\mathbb{E}[w^p_{cir}] = \alpha/(\alpha + \beta) = 1/I$, which agrees with the observation in Figure S3. Furthermore, since the Dirichlet distribution is a generalization of the beta distribution, we can conclude that the ionization efficiencies $(w^p_{c1r}, \ldots, w^p_{cIr})$ follow a Dirichlet distribution, as in Equation (6), with equal parameter values, $\alpha_1 = \cdots = \alpha_I$.

We further investigated the correlation between ionization efficiencies in two con-

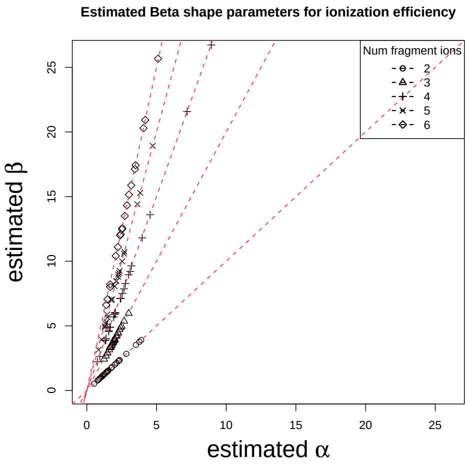

Figure S4: Scatter plot of the shape parameters α and β of the beta distribution fitted to the ionization efficiencies from the precursors with I fragment ions, for $I = 2, 3, \ldots, 6$, across 30 randomly selected proteins. The red dotted line represents the straight line with a slope of $(I - 1)$ and an intercept of zero.

ditions, w^p_{1ir} and w^p_{2ir}. Figure S5 illustrates the scatter plots of w^p_{1ir} and w^p_{2ir} from real MS experiments under the conditions "DMSO" and "100pM". The Pearson correlation coefficient is presented in the title of each panel. We conclude that the correlation is 0.72 in the case of $I = 2$ fragment ions, 0.89 in the case of $I = 3$, and it increases as the number of ions increases. This correlation is significantly high and can substantially affect the performance of classical statistical methods.

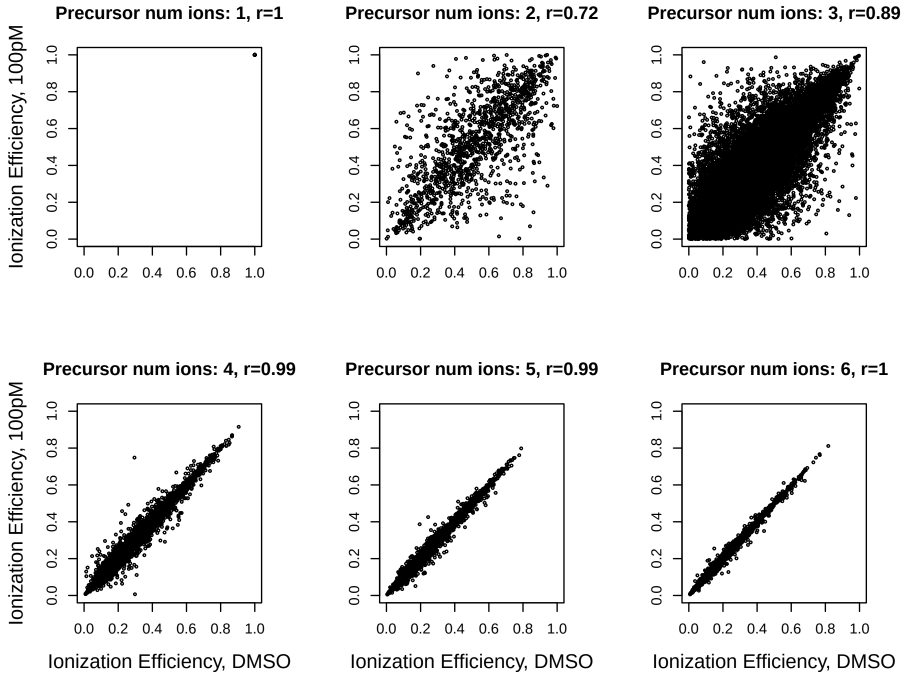

Figure S5: Scatter plots of ionization efficiencies between two conditions, "DMSO" and "100pM". The Pearson correlation coefficient is shown in the title of each panel.

S3 Classical Statistical Tests

Let μ_c^p denote the bias-corrected means of $\log_{10}$-transformed peptide quantities for conditions $c = 1, 2$, respectively; that is, $\mu_c^p = \tilde{\mu}_c^p - \log_{10} u_c^p$, $c = 1, 2$. For comparing the mean values, the mean difference between the two groups can be written as:

$$L = \mu_1^p - \mu_2^p. \tag{S1}$$

Equation (S1) can be extended to a linear combination of multiple mean values to address group comparisons involving two or more expected values, expressed as $L = \sum_c a_c \mu_c^p$ for any contrast coefficients a_c, $c = 1, \ldots, C$, satisfying $\sum_c a_c = 0$. In general, a test statistic for testing group differences, e.g., $H_0 : \mu_1^p = \mu_2^p$ versus $H_A : \mu_1^p \neq \mu_2^p$, can be expressed in the following form (Kohler et al., 2023):

$$t = \frac{\hat{L}}{\mathrm{SE}(\hat{L})}, \tag{S2}$$

where $\hat{L}$ is an estimate of L and $\mathrm{SE}(\hat{L})$ is the standard error of the estimate $\hat{L}$.

In this study, we used the independent samples t-test and the paired t-test as representatives of the two-step methods and feature-based methods, respectively.

(1) Independent samples t-test:

The test statistic of the independent samples t-test can be formulated as

$$t_{\text{ind}} = \frac{\hat{L}_{\text{ind}}}{\mathrm{SE}(\hat{L}_{\text{ind}})}, \tag{S3}$$

where

$$\hat{L}_{\text{ind}} = \bar{y}_1^p - \bar{y}_2^p, \tag{S4}$$

and $\bar{y}_c^p = \sum_{r=1}^{R_c} y_{cr}^p / R_c$ are the sample means of the log-transformed peptide quantities, with $y_{cr}^p = \log_{10} q_{cr}^p$. The standard error term in the denominator of (S3) is calculated, under the equal variance assumption, $\mathrm{Var}(y_{1r}^p) = \mathrm{Var}(y_{2r}^p)$, as follows:

$$\widehat{\mathrm{SE}}(\hat{L}_{\text{ind}}) = \sqrt{s^2_{\text{pooled}}(y^p) \cdot \left(\frac{1}{R_1} + \frac{1}{R_2}\right)}, \tag{S5}$$

where $s^2_{\text{pooled}}(y^p) = ((R_1 - 1) \cdot s^2(y_1^p) + (R_2 - 1) \cdot s^2(y_2^p))/(R_1 + R_2 - 2)$ is the pooled variance, with $s^2(y_1^p)$ and $s^2(y_2^p)$ being the sample variances of y_{1r}^p and y_{2r}^p, respectively. The null hypothesis is rejected at the significance level α if $|t_{\text{ind}}| > t_{\nu_{\text{ind}}}(\alpha)$, where the degrees-of-freedom ν_{ind} is computed as

$$\nu_{\text{ind}} = R_1 + R_2 - 2. \tag{S6}$$

(2) Paired t-test:

Let $x_{cir}^{p} = \log_{10} f_{cir}^{p}$ denote the log-transformed fragment ion quantities. The paired t-test assumes that the number of replicates is consistent across all conditions, i.e., $R_1 = R_2 \equiv R$, and that the differences in fragment ion quantities, $d_{ir}^{p} \equiv x_{1ir}^{p} - x_{2ir}^{p}$, are independent and identically distributed across fragment ions $i = 1, \ldots, I$ and replicates $r = 1, \ldots, R$. The test statistic is formulated as

$$t_{\text{paired}} = \frac{\hat{L}_{\text{paired}}}{\text{SE}(\hat{L}_{\text{paired}})}, \tag{S7}$$

where

$$\hat{L}_{\text{paired}} = \bar{\bar{d}}^{p}, \tag{S8}$$

and

$$\widehat{\text{SE}}(\hat{L}_{\text{paired}}) = \sqrt{\frac{s^2(d^p)}{IR}}, \tag{S9}$$

with $\bar{\bar{d}}^{p} = \sum_{i=1}^{I} \sum_{r=1}^{R} d_{ir}^{p}/(IR)$ representing the sample mean, and $s^2(d^p) = \sum_{i=1}^{I} \sum_{r=1}^{R} (d_{ir}^{p} - \bar{\bar{d}}^{p})^2/(IR-1)$ representing the sample variance of d_{ir}^{p}. The null hypothesis is rejected at the significance level α if $|t_{\text{paired}}| > t_{\nu_{\text{paired}}}(\alpha)$, where the degrees-of-freedom ν_{paired} is computed as

$$\nu_{\text{paired}} = IR - 1. \tag{S10}$$

S4 Theoretical Analysis

Proposition S1 explicitly expresses the variance term $\mathrm{Var}(\zeta^p)$ in terms of α_0^p and β_0^p.

Proposition S1. *Let $X \sim Beta(\alpha_0^p, \beta_0^p)$ be a random variable with a beta distribution, and let $Y = -\log X$ be the negative of its natural logarithmic transformation. Then, the expected value and variance of Y are given by*

$$\begin{aligned} \mathbb{E}[Y] &= -\psi(\alpha_0^p) + \psi(\alpha_0^p + \beta_0^p) \\ Var(Y) &= \psi'(\alpha_0^p) - \psi'(\alpha_0^p + \beta_0^p), \end{aligned} \tag{S11}$$

where $\psi(x) = d\log\Gamma(x)/dx = \Gamma'(x)/\Gamma(x)$ is the digamma function, and $\psi'(x) = d\psi(x)/dx$ is its first derivative.

Proof of Proposition S1. With $X = e^{-Y} \equiv g(Y)$, the probability density function of Y is

$$f_Y(y) = f_X(g(y))\left|\frac{\mathrm{d}g(y)}{\mathrm{d}y}\right| = \frac{\Gamma(\alpha_0^p + \beta_0^p)}{\Gamma(\alpha_0^p)\Gamma(\beta_0^p)} e^{-y\alpha_0^p}(1 - e^{-y})^{\beta_0^p - 1}, \qquad y \geq 0. \tag{S12}$$

The moment generating function (MGF) of Y is given by

$$\begin{aligned} M_Y(t) = \mathbb{E}[e^{Yt}] &= \int_0^\infty e^{yt} f_Y(y)\mathrm{d}y \\ &= \frac{\Gamma(\alpha_0^p + \beta_0^p)}{\Gamma(\alpha_0^p)\Gamma(\beta_0^p)} \int_0^\infty e^{-y(\alpha_0^p - t)}(1 - e^{-y})^{\beta_0^p - 1}\mathrm{d}y \\ &= \frac{\Gamma(\alpha_0^p + \beta_0^p)}{\Gamma(\alpha_0^p)\Gamma(\beta_0^p)} \cdot \frac{\Gamma(\alpha_0^p - t)\Gamma(\beta_0^p)}{\Gamma(\alpha_0^p + \beta_0^p - t)} \\ &= \frac{\Gamma(\alpha_0^p + \beta_0^p)}{\Gamma(\alpha_0^p)} \cdot \frac{\Gamma(\alpha_0^p - t)}{\Gamma(\alpha_0^p + \beta_0^p - t)}. \end{aligned} \tag{S13}$$

The first derivative of $M_Y(t)$, $M_Y'(t) = \mathrm{d}M_Y(t)/\mathrm{d}t$, can be expressed by

$$\begin{aligned} M_Y'(t) &= \frac{\Gamma(\alpha_0^p + \beta_0^p)}{\Gamma(\alpha_0^p)} \cdot \left(\frac{-\Gamma'(\alpha_0^p - t)\Gamma(\alpha_0^p + \beta_0^p - t) + \Gamma(\alpha_0^p - t)\Gamma'(\alpha_0^p + \beta_0^p - t)}{\Gamma^2(\alpha_0^p + \beta_0^p - t)}\right) \\ &= M_Y(t) \cdot \left(-\psi(\alpha_0^p - t) + \psi(\alpha_0^p + \beta_0^p - t)\right). \end{aligned} \tag{S14}$$

It follows that

$$\mathbb{E}[Y] = M_Y'(0) = -\psi(\alpha_0^p) + \psi(\alpha_0^p + \beta_0^p). \tag{S15}$$

The second derivative of $M_Y(t)$, $M''_Y(t) = \mathrm{d}M'_Y(t)/\mathrm{d}t$, is given by

$$\begin{aligned} M''_Y(t) &= M'_Y(t) \cdot (-\psi(\alpha_0^p - t) + \psi(\alpha_0^p + \beta_0^p - t)) + M_Y(t) \cdot \left(\psi'(\alpha_0^p - t) - \psi'(\alpha_0^p + \beta_0^p - t)\right) \\ &= M_Y(t) \cdot (-\psi(\alpha_0^p - t) + \psi(\alpha_0^p + \beta_0^p - t))^2 + M_Y(t) \cdot \left(\psi'(\alpha_0^p - t) - \psi'(\alpha_0^p + \beta_0^p - t)\right). \end{aligned} \tag{S16}$$

It follows that

$$\mathbb{E}[Y^2] = M''_Y(0) = (-\psi(\alpha_0^p) + \psi(\alpha_0^p + \beta_0^p))^2 + \psi'(\alpha_0^p) - \psi'(\alpha_0^p + \beta_0^p), \tag{S17}$$

and that

$$\mathrm{Var}(Y) = \mathbb{E}[Y^2] - (\mathbb{E}[Y])^2 = \psi'(\alpha_0^p) - \psi'(\alpha_0^p + \beta_0^p). \tag{S18}$$

□

The Proposition S1 implies that the variance of $\zeta_c^p = \log_{10} u_c^p = \log u_c^p / \log 10$ is proportional to $\psi'(\alpha_0^p) - \psi'(\alpha_0^p + \beta_0^p)$, which decreases to zero as α_0^p increases to infinity. Since α_0^p and β_0^p are unknown hyperparameters, the result in Proposition S1 is used only to calculate the theoretical true values in the following simulation studies.

S5 Supplementary Figures and Tables

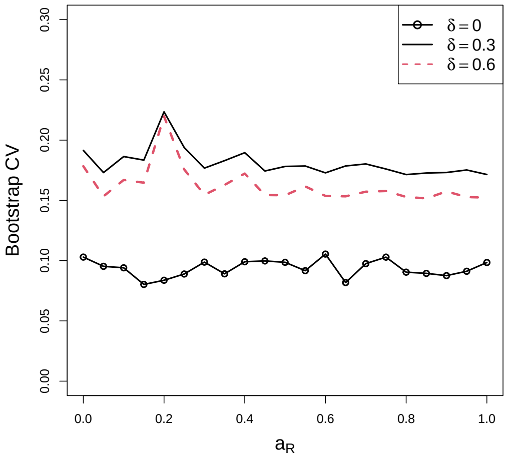

Figure S6: Coefficient of variation (CV) of the bootstrap test statistics for the proposed shrinkage-based method across different values of a_R, based on data sets generated under the conditions $\mu_1 = 5.0$ and $\mu_2 = 5.0 + \delta$ with $\delta = 0, 0.3, 0.6$.

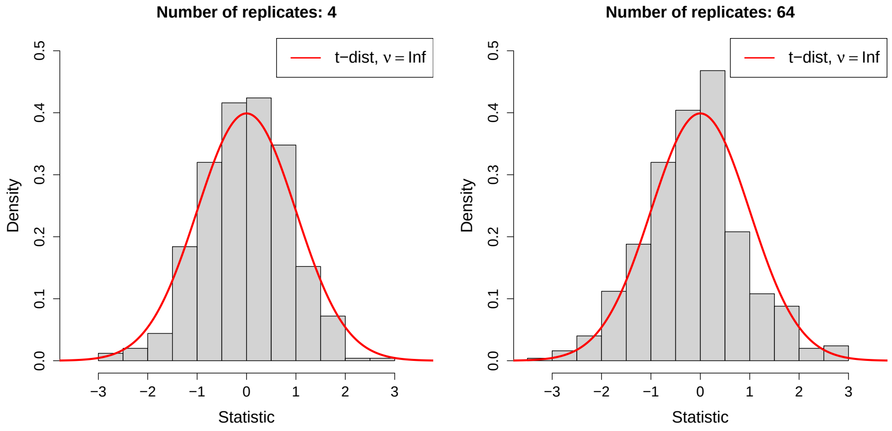

Figure S7: Empirical distribution of the shrinkage-based test statistic for $R = 4$ and $R = 64$. The red straight line represents the density function of the Student's t-distribution with $\nu = \infty$.

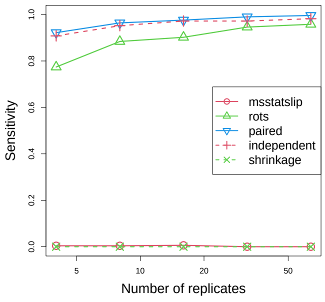

$(\alpha_0^p, \beta_0^p) = (11, 91)$, $\sigma_c^p = 0.05$

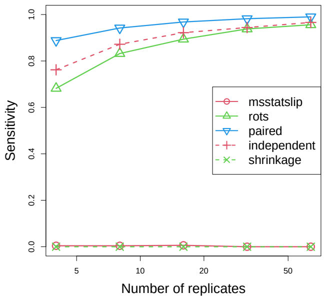

$(\alpha_0^p, \beta_0^p) = (11, 91)$, $\sigma_c^p = 0.1$

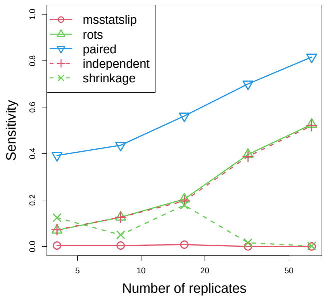

$(\alpha_0^p, \beta_0^p) = (11, 91)$, $\sigma_c^p = 0.8$

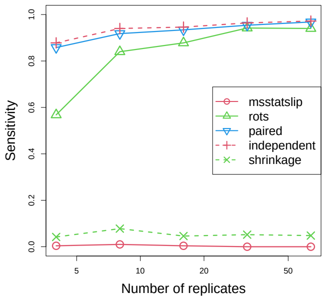

$(\alpha_0^p, \beta_0^p) = (4, 28)$, $\sigma_c^p = 0.05$

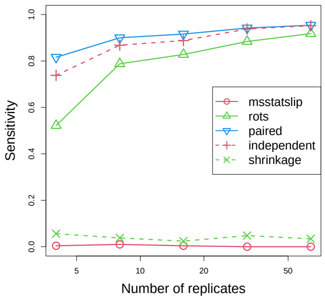

$(\alpha_0^p, \beta_0^p) = (4, 28)$, $\sigma_c^p = 0.1$

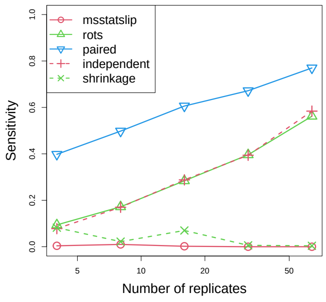

$(\alpha_0^p, \beta_0^p) = (4, 28)$, $\sigma_c^p = 0.8$

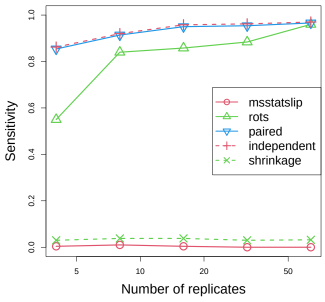

$(\alpha_0^p, \beta_0^p) = (2, 10)$, $\sigma_c^p = 0.05$

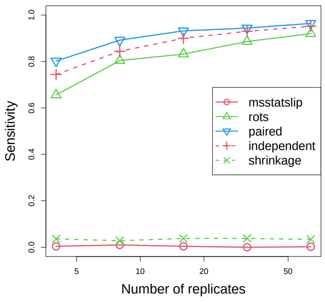

$(\alpha_0^p, \beta_0^p) = (2, 10)$, $\sigma_c^p = 0.1$

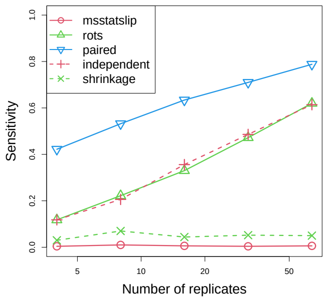

$(\alpha_0^p, \beta_0^p) = (2, 10)$, $\sigma_c^p = 0.8$

Figure S8: Sensitivity obtained using the five statistical testing methods: the MSstatsLiP, ROTS, paired t-test, independent samples t-test, and shrinkage t-test. The mean difference in log-transformed peptide quantities was set to $\delta = \log_{10}(2) \approx 0.3$.

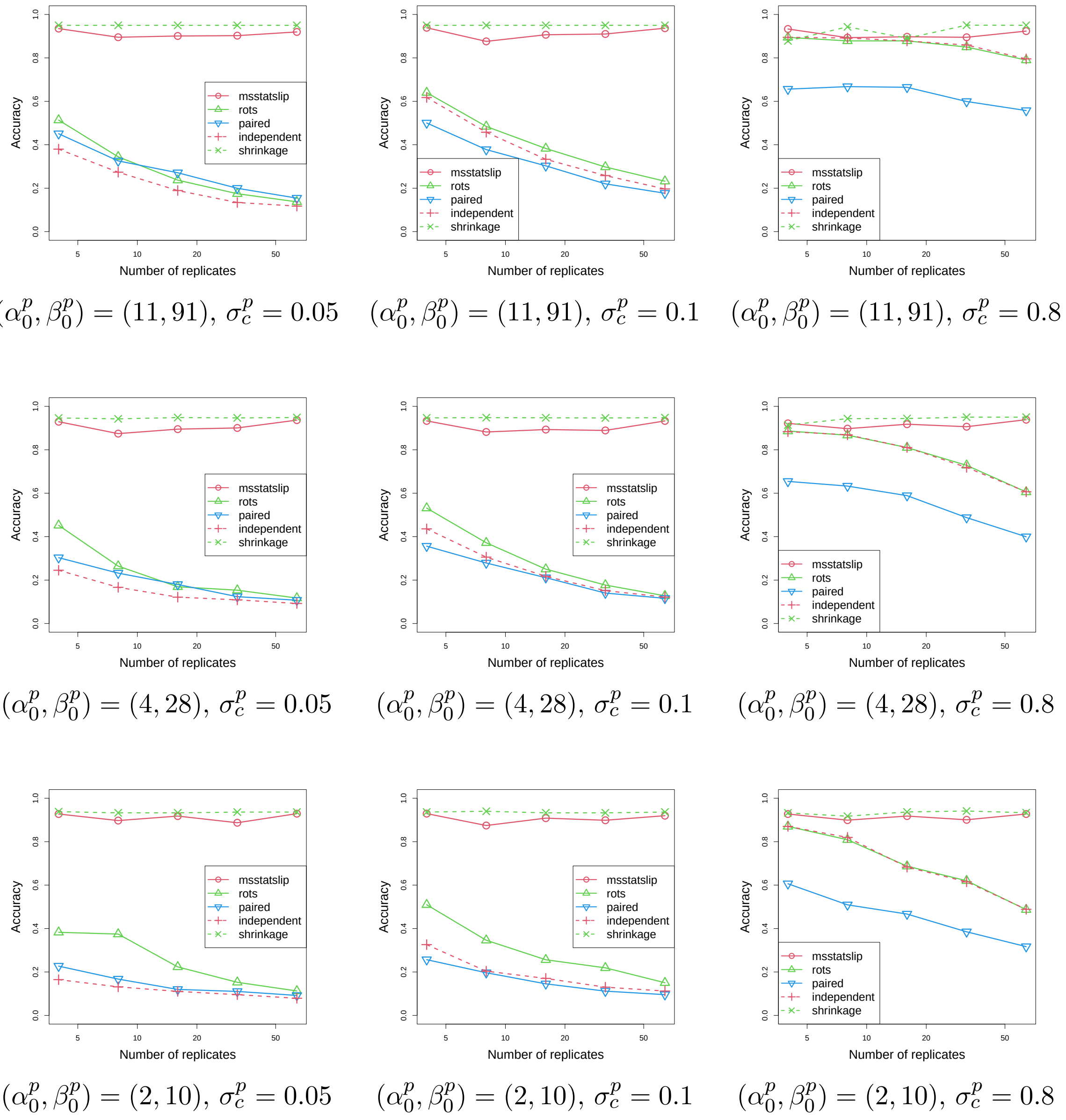

Figure S9: Accuracy obtained using the five statistical testing methods: the MSstatsLiP, ROTS, paired t-test, independent samples t-test, and shrinkage t-test. The proportion of peptides satisfying the alternative hypothesis was set to 5%, with a mean difference in log-transformed peptide quantities of $\delta = \log_{10}(2) \approx 0.3$.

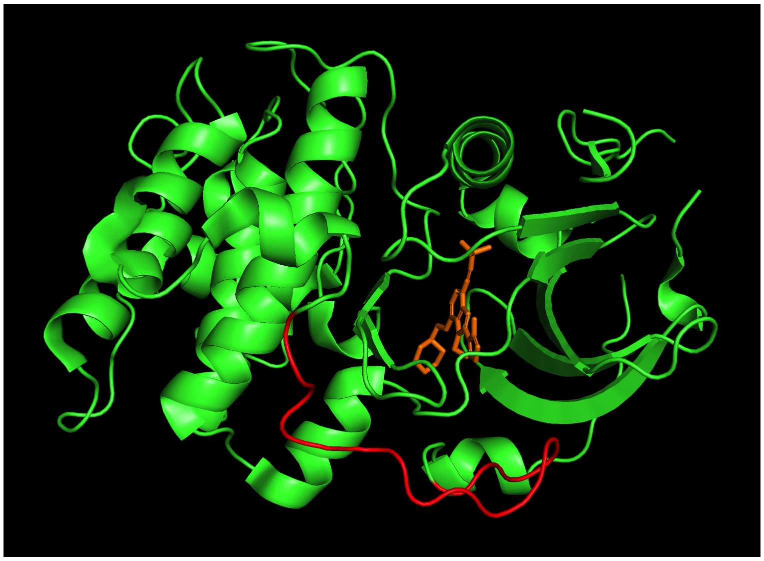

(a) AKT2

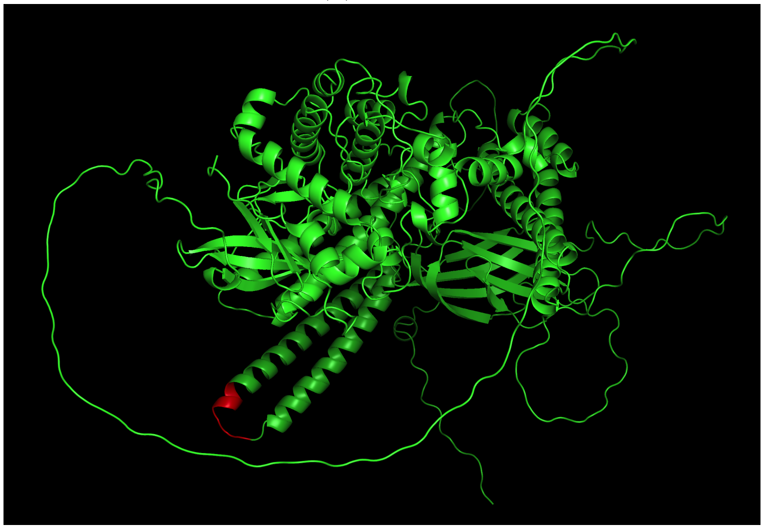

(b) PKN1

Figure S10: 3D structures of two kinase proteins, (a) AKT2 and (b) PKN1, with the detected peptides LLPPFKPQVTSEVDTR and ATTDLGR highlighted in red, respectively. For AKT2, the PDB database contains a a structure in which a drug named GSK690693 is bound to the protein (PDB ID: 3D0E). For PKN1, the 3D structure was obtained from AlphaFold (https://alphafold.ebi.ac.uk/entry/Q16512).

Table S2: Estimated values for $d = \mathrm{Var}(\zeta_c^p)$ obtained using the shrinkage t-test method.

	Comparison: DMSO/*						
	100pM	1nM	10nM	100nM	1uM	10uM	100uM
$\mathrm{Var}(\zeta_c^p)$	0.219	0.215	0.218	0.215	0.216	0.218	0.215

References

D. Kohler, M. Staniak, T.-H. Tsai, T. Huang, N. Shulman, O. M. Bernhardt, B. X. MacLean, A. I. Nesvizhskii, L. Reiter, E. Sabido, M. Choi, and O. Vitek. MSstats version 4.0: Statistical analyses of quantitative mass spectrometry-based proteomic experiments with chromatography-based quantification at scale. *Journal of Proteome Research*, 22(5):1466–1482, 2023. doi: 10.1021/acs.jproteome.2c00834.

I. Piazza, N. Beaton, R. Bruderer, T. Knobloch, C. Barbisan, L. Chandat, A. Sudau, I. Siepe, O. Rinner, N. de Souza, P. Picotti, and L. Reiter. A machine learning-based chemoproteomic approach to identify drug targets and binding sites in complex proteomes. *Nature Communications*, 11:4200, 2020. doi: 10.1038/s41467-020-18071-x.

M. West. On scale mixtures of normal distributions. *Biometrika*, 74(3):646–648, 1987. doi: 10.1093/biomet/74.3.646.
